# Characterization of Secondary Metabolites of Leaf Buds from Some Species and Hybrids of *Populus* by Gas Chromatography Coupled with Mass Detection and Two-Dimensional High-Performance Thin-Layer Chromatography Methods with Assessment of Their Antioxidant Activity

**DOI:** 10.3390/ijms25073971

**Published:** 2024-04-03

**Authors:** Loretta Pobłocka-Olech, Valery A. Isidorov, Mirosława Krauze-Baranowska

**Affiliations:** 1 Department of Pharmacognosy with Medicinal Plants Garden, Medical University of Gdańsk, 80-416 Gdańsk, Poland; krauze@gumed.edu.pl; 2 Institute of Forest Sciences, Bialystok Technical University, 15-351 Bialystok, Poland; isidorov@uwb.edu.pl

**Keywords:** poplar buds, bioautography, flavonoids, phenolics, standardization

## Abstract

Poplars provide medicinal raw plant materials used in pharmacy. Leaf buds are one of the herbal medicinal products collected from poplars, having anti-inflammatory and antiseptic properties, but there are no quality standards for their production and there is a need to determine their botanical sources. Therefore, the chemical compositions of the leaf buds from four species and varieties of poplars, *Populus balsamifera*, *P. × berolinensis*, *P. × canadensis* ‘Marilandica’, and *P. wilsonii* were investigated and compared using gas chromatography coupled with mass detection (GC-MS) and two-dimensional high-performance thin-layer chromatography (2D-HPTLC) in order to search for taxa characterized by a high content of biologically active compounds and with a diverse chemical composition that determines their therapeutic effects. The presence of 163 compounds belonging to the groups of flavonoids, phenolic acids derivatives, glycerides, and sesquiterpenes was revealed. Moreover, the conditions for the separation and identification of biologically active compounds occurring in analyzed leaf buds using 2D-HPTLC were optimized and used for metabolomic profiling of the studied poplars, enabling their fast and simple botanical identification. The total phenolic (TPC) and flavonoid (TFC) contents of examined extracts were determined and their antioxidant capacities were estimated by spectrophotometric DPPH, ABTS, and FRAP assays. Based on the analysis of phytochemicals and antioxidant activity, *P. × berolinensis* buds were selected as the raw plant material for medicinal purposes with the highest content of active compounds and the strongest antioxidant activity.

## 1. Introduction

Poplars are very common trees around the world belonging to the Salicaeae family. They grow in natural habitats but many of them are cultivated as a landscaping element or for commercial purposes [1]. The species of the *Populus* genus are characterized by fast growth and usually have low soil requirements, so they are an economically efficient source of renewable energy and biomass for the paper industry [2]. Because of this, in some countries (Germany, England, Italy, USA) they are cultivated as a short-rotation crop [1,3]. Poplars are valuable medicinal plants used in traditional and conventional systems for the treatment of diverse diseases [4]. Poplar buds (*Populi gemmae*) are one of three medicinal raw plant materials from the *Populus* genus characterized by anti-inflammatory and antiseptic properties, and they could be obtained as a by-product from the production of biomass for bioenergy purposes. Poplar ointment (*Unguentum Populeon*) was used in folk medicine to relieve skin inflammation, burns, and wounds, as well as muscle and rheumatic pain [5]. Currently, Gilead balm is available on the UK pharmacy market, containing a poplar bud preparation intended for both oral and topical use. Internally, it is used as an antibacterial and expectorant in chronic inflammation of the upper respiratory tract, externally for superficial skin injuries, and as a disinfectant in laryngitis [6]. However, the problem is determining the botanical source/origin of the raw plant material for its production. Some of the literature data indicate that balsam of Gilead is a tincture obtained from the buds of *Populus candicans* Ait. [7], but according to other sources, it contains preparations of leaves and flower buds of *P. nigra* L. or other species, including *P. candicans* Ait. and *P. balsamifera* L. [6].

*Populi gemmae*, due to its rich chemical composition, containing flavonoids, phenolic acids and their derivatives, and essential oil, is a valuable raw plant material with traditional use as an antiseptic and anti-inflammatory remedy [4]. The anti-inflammatory and antioxidant properties of the buds of some poplar species have been confirmed in a number of in vitro tests. The antioxidant activity could be involved in the anti-inflammatory mechanism of extracts obtained from various raw plant materials.

In vitro studies on murine macrophages of the RAW 264.7 cell line showed a significant anti-inflammatory effect of the ethanol extract from *P. × canadensis* buds (10 and 20 µg/mL) stimulated with LPS (200 ng/mL) in combination with IFN-γ (10 ng/mL) and in vivo studies (25 and 100 mg/kg) on LPS-induced endotoxemia (1 mg/kg for 3 h) and acute lung injury in mice [8]. The examined extract was standardized for the content of flavonoids (126.23 mg QE/g) and total phenolic compounds (145.54 mg GAE/g). HPLC analysis showed that flavonoids were dominated by chrysin, pinocembrin, and galangin (2.83–4.17 g/100 g of extract), whereas in the group of phenolic acids, often found in poplar buds, caffeic and *p*-coumaric acids were absent. Both studies demonstrated that *P. × canadensis* poplar bud extract has anti-inflammatory effects, inhibiting the secretion of specific inflammatory cytokines IL-6, IL-10, MCP-1, and TNF-α, and blocking the activation of nuclear factor (NF)-ĸB. Moreover, lung histopathology showed that pretreatment with poplar bud extract at a higher dose (100 mg/kg) inhibited LPS-induced changes in murine lung tissues [8].

Our previous in vitro studies on human gingival fibroblasts (HGF-1), pro-inflammatory stimulated by silver nanoparticles (3.5 µg/mL), showed the anti-inflammatory properties of extracts from *P. × berolinensis* (7.5 µg/mL) and *P. nigra* (15 µg/mL) buds by decreasing the IL-6 and IL-1β release in HGF-1 cells and the downregulation of mRNA for both cytokines [9]. The inhibition of pro-inflammatory cytokines was also demonstrated for two flavanones present in these extracts, inocembrin and pinostrobin (20 and 40 µM, respectively), in a dose-dependent manner. Moreover, the expression of COX-2 protein by pinocembrin and *P. × berolinensis* bud extract was demonstrated, which was distinguished from other extracts by its high flavanones content. These effects were not observed for buds from *P. lasiocarpa*, which did not contain flavonoids [9].

In vitro studies using LPS (100 ng/mL)-activated human leukemic monocytes THP-1 showed anti-inflammatory potential for the hydroalcoholic extracts from the buds of *P. nigra* (1.56 µg/mL), *P. deltoides* (3.12 µg/mL), and *P. trichocarpa* (6.25 µg/mL) [10]. The analyzed extracts differed in total phenolic content (*P. nigra* 134.02 mg GAE/g, *P. trichocarpa* 60.07 mg GAE/g, and *P. deltoides* 58.19 mg GAE/g), as well as in the presence of individual compounds identified by the HPLC-DAD-MS method, belonging to flavonoids and derivatives of phenolic acids, mainly caffeic and *p*-coumaric acids. Each of the tested poplar bud extracts inhibited the production of pro-inflammatory cytokines, such as tumor necrosis factor-a/TNF-a, interleukin-6/IL-6, and IL-1β, as well as the extracellular release of the high-mobility group box 1 (HMGB1), a “damage-associated molecular pattern” (DAMP) molecule, that is involved in the pathogenesis of various inflammatory diseases. However, in both antioxidant capacity assays used, ABTS and DPPH, the highest activity was demonstrated by the extract from *P. nigra* buds, characterized by a total phenolic content twice as high as the others and the presence of caffeic acid derivatives, e.g., caffeic acid phenylethyl ether (CAPE), pinocembrin, and pinobanksin butyric and acetic esters [10].

The free radical scavenging ability of dry aqueous extract from *P. nigra* buds was proven by oxygen radical absorbance capacity (ORAC) and cellular antioxidant activity (CAA) assay on normal human dermal fibroblasts (NHDF) [11]. HPLC-DAD analysis of the tested extract showed the presence of salicin, caffeic and *p*-coumaric acids, and pinobanksin as the dominant compounds and the total phenolic content was 180 mg caffeic acid equivalent/g. Studies using the NHDF cell line demonstrated the transcriptional effect of poplar bud extract which modulated the expression of the antioxidant enzyme catalase gene, chemokine (C-C motif) ligand 5 (CCL5) gene, Krupper-like factor 10 (KLF10), transcription factor E2F4, and EGF response factor 1 (ZFP36L1) genes involved in antioxidant defense, inflammatory response, and cell renewal [11].

Many other data have demonstrated the antioxidant effects of *P. nigra*, *P. balsamifera*, and *P. × canadensis* buds in various tests [8,12,13,14,15] and the dependence of their intensity on chemical composition; the presence of flavonoids and phenolic acids was especially observed. A number of studies have shown significant variability in the chemical composition of buds from different poplar species and varieties [16,17,18,19,20]; there is, therefore, a need to define/indicate *Populus* taxa that may be a source of medicinal raw material, valuable in terms of anti-inflammatory/antioxidant activity, and to develop methods for their standardization. Although poplar buds have monographs in the German Commission E and the British Herbal Compendium, they do not contain quality standards for this herbal product that correlate with its anti-inflammatory/antioxidant activity.

There are a lot of research data on the chemical composition of poplar buds [7,17,19,21,22], but many species and varieties remain unexplored, so there is a need to determine their composition using modern analytical methods. GC-MS is most often used in the analysis of the chemical composition of poplar buds because it allows one separation to identify most of the compounds from various chemical groups—polyphenols, tri- and sesquiterpenes, aliphatic acids, alcohols, etc.—present in these plant matrices [16,17,19,22,23].

The aim of the research was to recognize the chemical composition of the buds of poplar trees often grown in Poland and determine their antioxidant capacity in order to select taxa with the richest chemical compositions and highest antioxidant activities as sources of raw material for medicinal purposes.

Therefore, our research was focused on the analysis of the chemical composition of leaf buds collected from four species and varieties of poplars cultivated in Poland, namely *Populus balsamifera*, *P. × berolinensis*, *P. × canadensis* ‘Marilandica’, and *P. wilsonii*, using gas chromatography coupled with mass detection (GC-MS). Moreover, the conditions for the analysis of biologically active compounds from the poplar buds were optimized using two-dimensional high-performance thin-layer chromatography (2D-HPTLC), which is relatively fast and cheap compared to GC-MS, enabling the identification of their botanical origin by comparing the obtained chromatographic metabolomic profiles.

Moreover, the tested poplar bud extracts were quantitatively characterized by determining the total flavonoid (TFC) and total phenolic (TPC) content, and their antioxidant capacity was assessed using 2D-HPTLC bioautography and spectrophotometric determinations using DPPH (2,2-diphenyl-1-picrylhydrazyl), ABTS (2,2′-azino-bis(3-ethylbenzothiazoline-6-sulfonic acid) diammonium salt), and FRAP (ferric reducing antioxidant power) assays.

## 2. Results

### 2.1. GC-MS Analysis

The chemical compositions of the leaf buds of four species and varieties of poplars occurring in Poland were examined using the GC-MS method, namely *Populus balsamifera*, *P.* × *berolinensis*, *P.* × *canadensis* ‘Marilandica’, and *P. wilsonii*.

GC-MS studies enabled the identification of a total of one hundred and sixty-three compounds occurring in the analyzed bud extracts, primarily from the group of flavonoids, phenolic acids and their derivatives, as well as glycerides and terpenes. There were significant differences in the chemical compositions of the tested leaf buds of different species and varieties of the *Populus* genus (Table 1 and Table S1).

**Figure 1:**
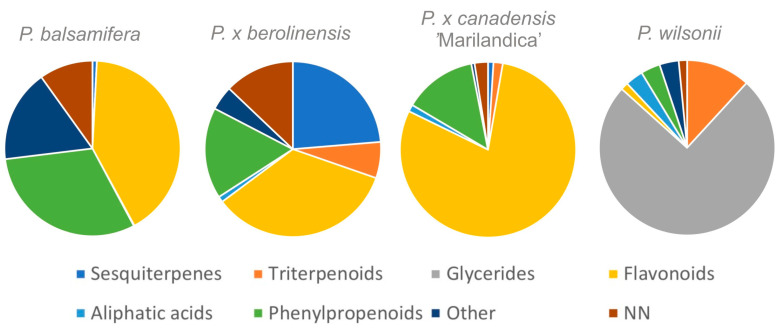
The content (% of TIC) of main group of compounds identified by GC-MS method in analyzed poplar buds.

The leaf buds of *P. balsamifera*, *P. × berolinensis*, and *P. × canadensis* ‘Marilandica’ were characterized by a high content of flavonoid compounds (41.28, 34.44, and 79.51% TIC (total ion current) respectively) and phenylpropanoids (30.85, 16.82, and 13.43% TIC, respectively), in contrast to *P. wilsonii* buds (1.38 and 3.57% TIC, respectively), which were distinguished by the presence of phenylpropanoid glycerides as the dominant ones (74.94% TIC). Moreover, the buds of *P. × berolinensis*, unlike the other analyzed taxa, contained more sesquiterpenes (23.68% TIC) (Figure 1, Table 1).

A total of forty flavonoids were identified, of which pinocembrin from the flavanone group, galangin from the flavonols, and pinobanksin and its acetate from the flavanonols were present in all tested extracts. Flavonoids were the dominant group of compounds in *P.* × *canadensis* ‘Marilandica’ (79.51% TIC). This variety was characterized by the most diverse set of flavonoids (29 compounds identified), with the predominance of galangin (18.59% TIC), chrysin (12.38% TIC), pinobanksin (12.55% TIC) and its acetate (11.78% TIC), and pinocembrin (7.86% TIC) (Table 1).

*P. × berolinensis* buds were characterized by a high content of the following flavanones: pinocembrin (13.18% TIC), pinostrobin (6.85% TIC), pinobanksin 3-acetate (4.12% TIC), and galangin (3.58% TIC). The buds of *P. balsamifera* were distinguished by the presence of chalcones in high concentrations, including 2′,6′-dihydroxy-4′-methoxydihydrochalcone (5.52% TIC), 2′,6′-dihydroxy-4′-methoxychalcone (pinostrobin chalcone) (3.66% TIC), and 2′,4′,6′-trihydoxydihydrochalcone (2.51% TIC). The presence of pinostrobin chalcone, 2′,4′,6-trihydroxydihydrochalcone, and 2′,6′,α-trihydroxy-4′-methoxychalcone was detected for the first time in the buds of *P. × berolinensis* and *P. × canadensis* ‘Marilandica’. In the last of the varieties mentioned, the presence of 2′,6′-dihydroxy-4′-methoxydihydrochalcone was also found (Table 1).

Thirty-one compounds were identified in the group of phenolic acids and their derivatives. All tested poplar extracts contained caffeic, *p*-coumaric, and benzoic acid (Table 1). *P. balsamifera* buds were characterized by a high content of *p*-coumaric acid (15.36% TIC), whereas the remaining analyzed poplar taxa were characterized by a high content of caffeic acid (from 2.49 to 4.92% TIC). Moreover, *P. × berolinensis* extracts contained a significant content of 3,4-dimethoxycinnamic acid (3.65% TIC) and isoferulic acid (1.95% TIC) (Table 1).

The greatest number of cinnamic acid derivatives were determined in the buds of *P. balsamifera*, including (*E*)-*p*-cinnamyl coumarate (7.74% TIC), 2-methyl-2-butenyl(*E*)-*p*-coumarate (4.68% TIC), isopentyl *p*-coumarate (4.31% TIC), 2-phenylethyl *p*-coumarate (1.88% TIC), and isobutyl *p*-coumarate (1.31% TIC).

In extracts from *P. × berolinensis* and *P. × canadensis* ‘Marilandica’, the group of phenolic acid derivatives was dominated by caffeic acid phenethyl ester (CAPE) (1.39 and 1.55% TIC), next to 2-methyl-2-(*E*)-butenyl caffeate (1.11% TIC) in *P. × berolinensis* and 3-methyl-2-butenyl (*E*)-caffeate (2.46% TIC) in *P. × canadensis* ‘Marilandica’.

A high content of sesquiterpenes was found in the buds of *P. × berolinensis* (23.68% TIC), the dominant ones being hinesol (9.0% TIC) and β-eudesmol (5.99% TIC), next to cubenol (1.96% TIC) and acorenol (1.88% TIC). Additionally, α-copaen-11-ol, 1-epi-cubenol, agaraspirole, α-muurolene, α-calacorene, α-terpineol, and α-ylangen were identified in this hybrid (Table 1). In the buds of *P. × canadensis* ‘Marilandica’, the presence of α-cadinol, γ- and β-eudesmols, δ-cadinene, ar-curcumene, α-muurolene, α-calacorene, and acorenol were demonstrated. *P. balsamifera* buds contained γ-(0.37% TIC) and ar-curcumenes (0.24% TIC), sesquicineole (0.19% TIC), and trace amounts of β-copaene, α-bisabolol, and β-bisabolene. However, only trace amounts of β- and γ-eudesmols were found in *P. wilsonii* extracts. Additionally, ar-curcumen and acorenol were identified in this material.

Glycerol derivatives occurred only in the plant material of *P. wilsonii* and constituted the dominant group of compounds in this species. The highest concentrations were determined for 2-acetyl-1-*p*-coumaroyl-3-caffeoylglycerol (lasiocarpin B) (27.16% TIC), 2-acetyl-1-caffeoyl-3-feruloylglycerol (25.87% TIC), and 1, 3-di-*p*-coumaroyl-2-acetylglycerol (lasiocarpin A) (16.73% TIC) (Table 1).

A relatively high concentration of triterpenoids (11.76% TIC) was found in the analyzed extracts of *P. wilsonii*, in which the highest concentration was dipterocarpol (4.32% TIC), whereas the concentrations of lanosterol (0.53% TIC), α- and β-amyrin (0.22 and 0.10% TIC), and triterpenol (0.08% TIC) were low. Moreover, it is worth noting that the presence of palmitic and α-linolenic acid was found in all tested poplar buds.

### 2.2. 2D-HPTLC Analysis

**Figure 2:**
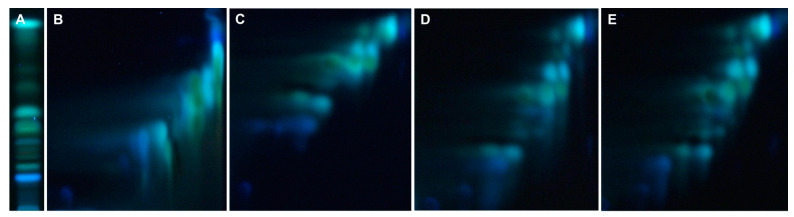
The 1D and 2D HPTLC separation of extract from the buds of *Populus balsamifera* (Bl) obtained using HPTLC silica gel F_254_, (**A**) chloroform:methanol:formic acid (88:7:5, *v*/*v*/*v*); (**B**) first direction (1D): n-hexane:ethyl acetate:acetic acid (64:28:10, *v*/*v*/*v*), second direction (2D): chloroform:methanol:formic acid (88:7:5, *v*/*v*/*v*); (**C**) 1D: chloroform:methanol:formic acid (88:7:5, *v*/*v*/*v*); 2D: n-hexane:ethyl acetate:acetic acid (64:28:10, *v*/*v*/*v*), and 1D: chloroform:methanol:formic acid (95:2:3, *v*/*v*/*v*); (**D**) 2D: n-hexane:ethyl acetate:acetic acid (64:28:10, *v*/*v*/*v*), (**E**) 2D: n-hexane:ethyl acetate:formic acid (60:40:1.3, *v*/*v*/*v*), detection: 2% AlCl_3_, λ—366 nm.

The developed 2D-HPTLC separation conditions included the use of high-performance silica gel as an adsorbent and a mixture of chloroform:methanol:formic acid (95:2:3, *v*/*v*/*v*) as the mobile phase for the first direction (1D) and a mixture of n-hexane:acetate ethyl:formic acid (60:40:1.3, *v*/*v*/*v*) in the second (2D) (Figure 2E).

HPTLC plates were derivatized with a 2% methanol solution of AlCl_3_ or 0.5% solution of anisaldehyde in a 5% methanol solution of sulfuric acid (95 °C, 3 min) and analyzed under UV light at λ—366 nm or under visible light (λ—500 nm). Table 2 shows the retention parameters for the reference substances used and the colors of their zones obtained after derivatization with different spraying reagents.

**Figure 3:**
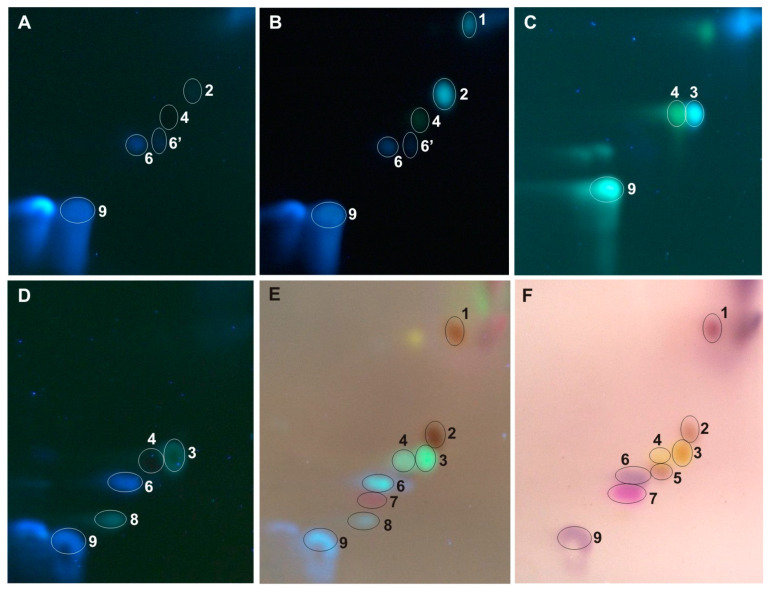
The 2D HPTLC separation of standard mixture: (**A**,**D**) UV λ—366 nm; (**B**,**C**) 2% AlCl_3_, λ—366 nm, (**E**) 0.5% anisaldehyde in 5% H_2_SO_4_ in MeOH (95 °C, 3 min), λ—366 nm, (**F**) 0.5% anisaldehyde in 5% H_2_SO_4_ in MeOH (95 °C, 3 min), λ—500 nm; **1**—pinostrobin, **2**—pinocembrin, **3**—galangin, **4**—chrysin, **5**—pinobanksin, **6**,**6′**—phenethyl ester of caffeic acid (CAPE), **7**—*p*-coumaric acid, **8**—kaempferol, **9**—caffeic acid.

**Figure 4:**
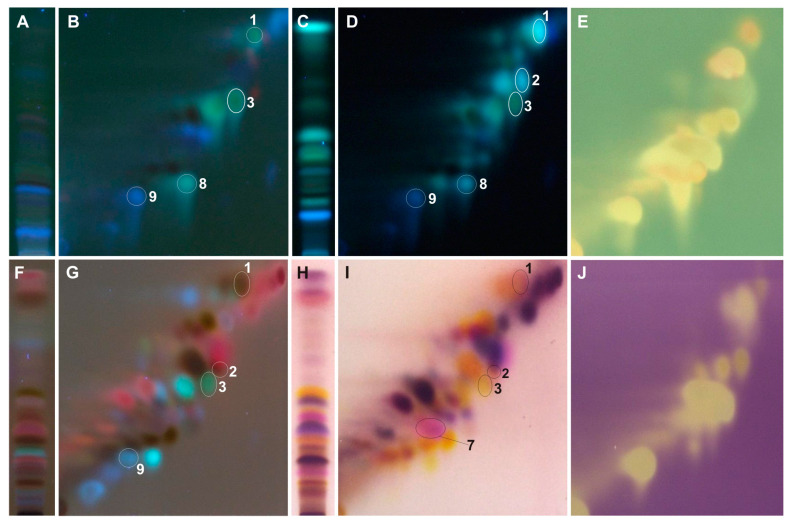
The 1D and 2D HPTLC separation of extract from the buds of *Populus balsamifera* (Bl) obtained using (**A**,**B**) UV λ—366 nm; (**C**,**D**) 2% AlCl_3_, λ—366 nm; (**F**,**G**) 0.5% anisaldehyde in 5% H_2_SO_4_ in MeOH (95 °C, 3 min), λ—366 nm; (**H**,**I**) 0.5% anisaldehyde in 5% H_2_SO_4_ in MeOH (95 °C, 3 min), λ—500 nm and bioautograms (**E**) ABTS and (**J**) DPPH; **1**—pinostrobin, **2**—pinocembrin, **3**—galangin, **7**—*p*-coumaric acid, **8**—kaempferol, **9**—caffeic acid.

**Figure 5:**
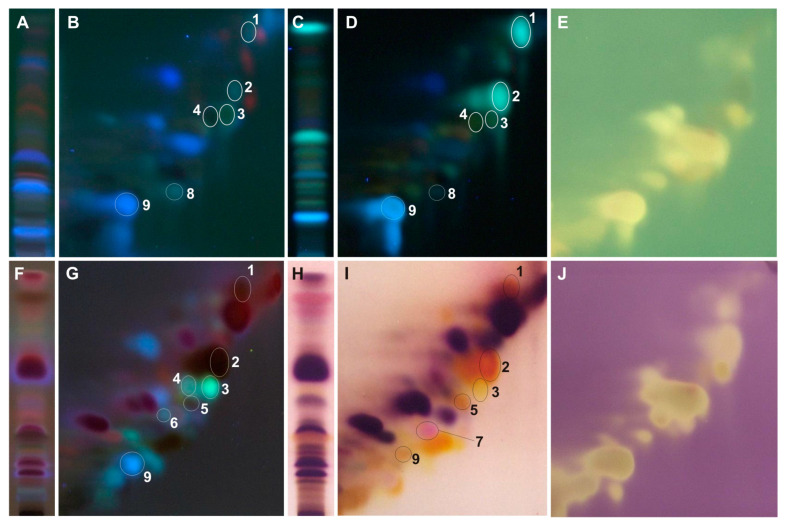
The 1D and 2D HPTLC separation of extract from the buds of *P. × berolinensis* (Br) obtained using (**A**,**B**) UV λ—366 nm; (**C**,**D**) 2% AlCl_3_, λ—366 nm; (**F**,**G**) 0.5% anisaldehyde in 5% H_2_SO_4_ in MeOH (95 °C, 3 min), λ—366 nm; (**H**,**I**) 0.5% anisaldehyde in 5% H_2_SO_4_ in MeOH (95 °C, 3 min), λ—500 nm and bioautograms (**E**) ABTS and (**J**) DPPH; **1**—pinostrobin, **2**—pinocembrin, **3**—galangin, **4**—chrysin, **5**—pinobanksin, **6**—phenethyl ester of caffeic acid (CAPE), **7**—*p*-coumaric acid, **8**—kaempferol, **9**—caffeic acid.

**Figure 6:**
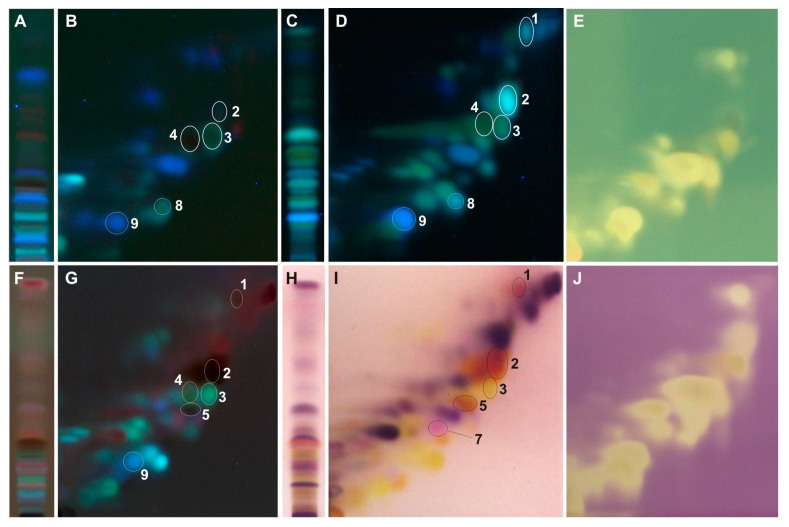
The 1D and 2D HPTLC separation of extract from the buds of *P. × canadensis* ‘Marilandica’ (Mr) obtained using (**A**,**B**) UV λ—366 nm; (**C**,**D**) 2% AlCl_3_, λ—366 nm; (**F**,**G**) 0.5% anisaldehyde in 5% H_2_SO_4_ in MeOH (95 °C, 3 min), λ—366 nm; (**H**,**I**) 0.5% anisaldehyde in 5% H_2_SO_4_ in MeOH (95 °C, 3 min), λ—500 nm and bioautograms (**E**) ABTS and (**J**) DPPH; **1**—pinostrobin, **2**—pinocembrin, **3**—galangin, **4**—chrysin, **5**—pinobanksin, **7**—*p*-coumaric acid, **8**—kaempferol, **9**—caffeic acid.

**Figure 7:**
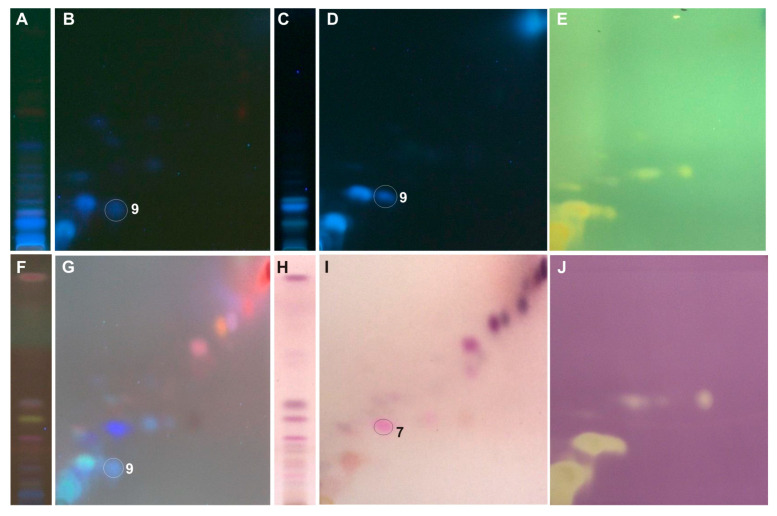
The 1D and 2D HPTLC separation of extract from the buds of *P. wilsonii* (Ws) obtained using (**A**,**B**) UV λ—366 nm; (**C**,**D**) 2% AlCl_3_, λ—366 nm; (**F**,**G**) 0.5% anisaldehyde in 5% H_2_SO_4_ in MeOH (95 °C, 3 min), λ—366 nm; (**H**,**I**) 0.5% anisaldehyde in 5% H_2_SO_4_ in MeOH (95 °C, 3 min), λ—500 nm and bioautograms (**E**) ABTS and (**J**) DPPH; **7**—*p*-coumaric acid, **9**—caffeic acid.

The developed 2D-HPTLC method enables the identification of caffeic and *p*-coumaric acid in all tested extracts (Figure 3, Figure 4, Figure 5, Figure 6 and Figure 7). Moreover, from the group of phenolic acid derivatives, caffeic acid phenethyl ester (CAPE) was detected in the *P. berolinensis* bud extract (Figure 5G). Among the flavonoids, in the buds of *P. balsamifera*, *P. × berolinensis*, and *P. × canadensis* ‘Marilandica’, the presence of galangin, pinocembrin, and pinostrobin was confirmed. Pinobanksin and chrysin were also identified in the latter two taxa, whereas kaempferol was detected in *P. balsamifera* and *P. × canadensis* ‘Marilandica’.

The developed 2D-HPTLC method was used to assess the antioxidant activity of the compounds from the tested poplar buds by bioautography using ABTS and DPPH solutions (Figure 4E,J, Figure 5E,J, Figure 6E,J and Figure 7E,J). It was found that the compounds showing antioxidant activity in the obtained bioautograms were primarily flavonoids and phenolic acids and their derivatives. The buds of *P. balsamifera*, *P. × berolinensis*, and *P. × canadensis* ‘Marilandica’ contained numerous antioxidant substances, in contrast to the buds of *P. wilsonii*.

### 2.3. Antioxidant Capacity

The antioxidant capacity of the analyzed poplar bud extracts was estimated using DPPH, ABTS, and FRAP spectrophotometric tests and was expressed in mM Trolox (6-hydroxy-2,5,7,8-tetramethylchroman-2-carboxylic acid—TEA) per 1 g of dry weight (d.w.) of the tested plant material (Table 3).

The highest capacity in the DPPH and FRAP tests was determined for the methanol extract from *P. × berolinensis*—7.5728 and 24.675 mM TEA/g, respectively. Only in the ABTS study, the *P. balsamifera* extract (12.1 mM TEA/g) showed a slightly greater ability to scavenge free radicals. In turn, the lowest values of antioxidant potential in the DPPH and ABTS tests were recorded for the *P. wilsonii* bud extract—2.4102 and 3.652 mM TEA/g, respectively.

### 2.4. Total Flavonoid (TFC) and Total Phenolic (TPC) Content

The total flavonoid content (TFC) was determined using a 1% solution of aluminum chloride (AlCl_3_) as a color reagent and expressed in rutin equivalents (RE). A very wide range of TFC was found in the tested buds of four poplar taxa, ranging from 7.92 mg/g in *Populus wilsonii* to 65.01 mg/g in *P.* × *berolinensis* (Table 3). In the buds of the remaining studied poplars, *P. balsamifera* and *P.* × *canadensis* ‘Marilandica’, relatively high contents of flavonoids were revealed—44.76 and 55.17 mg/g, respectively. No statistically significant differences were found between the TFC of *P.* × *berolinensis* and *P.* × *canadensis* ‘Marilandica’ and between the TFC of *P. balsamifera* and *P.* × *canadensis* ‘Marilandica’ (Table 3).

The total phenolic content (TPC) was determined using the Folin–Ciocalteu reagent and expressed in gallic acid equivalents (GAE). The highest concentration of phenols was found in the buds of *P.* × *berolinensis* (183.18 mg/g) and *P. balsamifera* (166.17 mg/g) (statistically insignificant difference *p* < 0.05). The lowest TPC was detected in the buds of *P. wilsonii* (64.96 mg/g) (statistically significant differences compared to TFC in the other tested poplar buds, *p* < 0.05) (Table 3).

## 3. Discussion

### 3.1. GC-MS Analysis

The results of phytochemical tests conducted in this study confirmed previous literature data on significant differences in the chemical composition of buds of various *Populus* species and their hybrids (Figure 1, Table 1 and Table S1). So far, detailed phytochemical studies have included poplar buds originating mainly from species and varieties found in North America and Asia [18,21,22,24]. Our research focused on identifying the chemical composition of buds from poplars grown in Europe. They confirm the need to select poplar buds in order to obtain medicinal raw plant materials with predictable and repeatable therapeutic effects, including anti-inflammatory ones.

Based on the data obtained from performed chromatographic analyses, the chemical composition of *P. wilsonii* buds was recognized for the first time [25], revealing the presence of phenylpropanoid glycerides as the dominant compounds (74.94% TIC), as well as several compounds from the triterpene group (11.76% TIC), phenolic acids (3.57% TIC), aliphatic acids (3.28% TIC), and flavonoids (1.38% TIC). Moreover, knowledge about the chemical composition of *P. × berolinensis* and *P. × canadensis* ‘Marilandica’ buds was significantly expanded. So far, only the presence of some flavonoid aglycones has been described in these raw plant materials based on TLC analyses [9,20,26]. Furthermore, γ- and β-eudesmols, α-cadinol, α-calacorene, acorenol, α-muurolene, and ar-curcumene were identified as components of the essential oil from leaf buds of an undescribed variety of *P. × canadensis* [16]. As a result of the research carried out, a number of compounds from the group of sesquiterpenes (23.68% TIC), cinnamic acids and their derivatives (16.82% TIC), triterpenes (6.66% TIC), and chalcones were identified in the buds of *P. × berolinensis.* In the buds of *P. × canadensis* ‘Marilandica’ phenylpropenoids (13.43% TIC), triterpenoids (1.71% TIC), aliphatic acids (1.29% TIC), and some new flavonoids and chalcones were detected, not previously described in this taxon. In turn, in the examined buds of *P. balsamifera*, the presence of several benzoic acid esters (3.04% TIC) and several new derivatives of *p*-coumaric acid (10.98% TIC) and caffeic acid, in addition to sesquicineol from sesquiterpene group, were found for the first time. The presence of flavonoids, chalcones, other phenylpropanoids, and sesquiterpenes previously described in this taxon was also confirmed (Table 1) [17,27,28,29].

In most of the analyzed buds, the dominant group was flavonoids (34.44–79.51% TIC), including chalcones, with the exception of *P. wilsonii* buds, which were characterized by a low content of these compounds (1.38% TIC). Galangin, pinocembrin, chrysin, pinobanksin, and its acetate were identified in the flavonoid set of *P. wilsonii* buds. In addition to the flavonoids previously described in the literature [26], pinobanksin, sakuranetin, isosakuranetin, and kaempferol were also detected in *P. × berolinensis* buds. The largest number of flavonoid compounds (29 compounds) was found in the leaf buds of *P. × canadensis* ‘Marilandica’. Among them, pinobanksin and its 3-isobutanoate, 3-butanoate, 5-pentenoate, and 3-hydrocinnamate derivatives, pinostrobin, 2′,6′,4-trihydroxy-4-methoxyflavone, sakuranetin, kaempferol, isorhamnetin, 3-methyl- and 3,4′-dimethyl- ethers of quercetin were identified in the buds of this variety for the first time. In the analyzed buds of *P. balsamifera*, acacetin was identified as a new flavonoid in the set of flavonoid compounds of this species. Flavonoid compounds such as galangin, pinocembrin, pinobanksin, and its acetate were revealed in all analyzed poplar buds.

All the above-mentioned flavonoid compounds—chrysin, pinocembrin, galangin, and pinobanksin—have demonstrated anti-inflammatory activity in a number of in vivo and in vitro tests [30,31,32,33,34,35]. In a rat model, galangin reduced collagen II and aggrecan degradation via the inhibition of Akt phosphorylation and NF-κB activation in chondrocytes and could reduce cartilage degradation after intra-articular injection. Studies on human rheumatic arthritis fibroblast-like synoviocytes (RAFLSs) stimulated by LPS showed that galangin protected cells by downregulating the NF-κB/NLRP3 signaling pathway. The tested compound inhibited the inflammatory response by reducing the levels of IL-1β, TNF-α, and IL-6 and suppressing the PI3K/AKT signal [31,32]. Galangin has shown protective potential on human dermal fibroblasts stimulated with hydrogen peroxide via inhibiting NF-кB activation, reducing inflammatory cytokine levels, and inducing the expression of IGF1R/Akt-related proteins [31].

Pinocembrin treatment significantly inhibited TNF-a-induced phosphorylation and degradation of the NF-ĸB inhibitor IĸBα in human chondrocytes [33]. Treatment of adjuvant-induced arthritis (AIA) in mice with pinocembrin reduced symptoms of arthritis, including amelioration of swelling, the degree of redness of the hind paw, and decreased range of motion. At the pathological level, this compound significantly reduced joint erosions and the percentages of infiltrating inflammatory cells [34].

Chrysin exerted a protective effect on human osteoarthritis chrondrocytes by suppressing high-mobility chromosomal protein (HMGB1). It increased the expression of the gene encoding the alpha-1 chain of type II collagen (COL2A1), while inhibiting cell apoptosis, MMP-13, and IL-6 [35]. Another study showed that chrysin significantly blocked IL-1β-stimulated IκB-α degradation and NF-κB activation in vitro using IL-1β-injured human chondrocytes. This compound inhibits IL-1β-induced NO and PGE2 production in human chondrocytes and downregulates the expression of COX-2 and iNOS [35].

Taking into account the above data and the significant content of pinocembrin, galangin, and chrysin in the buds of *P. × berolinensis* and *P. × canadensis* ‘Marilandica’, it can be assumed that these compounds will have a significant impact on their anti-inflammatory effect.

Chalcones are a relatively rare group of flavonoid compounds occurring in the plant kingdom, and their presence has so far been demonstrated in the buds of many poplar species, including *P. balsamifera*, *P. ×candicans*, *P. deltoides*, *P. koreana*, *P. laurifolia*, *P. szechuanica*, or *P. suaveolens*, etc. [7,18,19,22,36,37].

Among the analyzed poplar buds, the presence of five chalcones was confirmed in *P. balsamifera*, and for the first time, pinostrobin chalcone, 2′,4′,6-trihydroxydihydrochalcone, and 2′,6′,α-trihydroxy-4′-methoxychalcone were confirmed in the buds of *P. × berolinensis* and *P. × canadensis* ‘Marilandica’. Moreover, 2′,6′-dihydroxy-4′-methoxydihydrochalcone was detected in the latter variety (Table 1).

Hydroxycinnamoylated dihydrochalcone derivatives, called balsacones A-M, which were isolated from the buds of Canadian *P. balsamifera*, showed significant antimicrobial potential against gram (+) *Staphylococcus aureus* with MIC values ranging from 3.1 to 6.3 µM, but were inactive against Gram-negative *Escherichia coli*. The antibacterial effect of balsacones on Gram-positive bacteria is probably related to the presence of the 4-hydroxycinnamyl group of the A ring in the chemical structure of these compounds because compounds without this residue are inactive [29,38,39].

The confirmed high content of chalcones (14.5% TIC) in the flavonoid set from *P. balsamifera* buds (41.28% TIC) distinguishes this species from others and is of chemotaxonomic importance.

The GC-MS analyses showed the presence of caffeic, *p*-coumaric, and benzoic acids in all tested poplar buds. The buds of *P. balsamifera* were characterized by a high content of *p*-coumaric acid, whereas the remaining analyzed poplar taxa were characterized by a high content of caffeic acid and a significant content of 3,4-dimethoxycinnamic acid and isoferulic acid in *P. × berolinensis* (Table 1).

In vitro studies of the antioxidant properties of polyphenolic compounds identified in *Populus nigra* buds using the ORAC test showed higher activity of caffeic and *p*-coumaric acids compared to flavanone (pinocembrin) and flavanol aglycones (pinobanksin and its methyl derivative) and other tested phenolic acids (ferulic, isoferulic, and cinnamic). The observed differences in the activity of these compounds are related to the number and position of free hydroxyl groups in the aromatic ring. It was found that substituting one of the hydroxyl groups in the phenol ring reduces antioxidant activity, whereas the absence or blocking of all hydroxyl groups deprives this activity [11].

Phenolic acids, known for their antioxidant activity, are next to flavonoids, another group of secondary metabolites that may contribute to the anti-inflammatory effect of poplar bud extracts.

An interesting group of compounds detected in the studied poplar buds are phenylpropanoids. So far, the occurrence of phenolic acid esters in poplar buds, especially caffeic, cinnamic, and coumaric acid derivatives, has been described for many species and hybrids of this genus [17,18,19,21,40]. However, the GC-MS analyses carried out for the first time also confirm their presence in the buds of *P.* × *berolinensis*, *P.* × *canadensis* ‘Marilandica’, and *P. wilsonii*. This group of compounds in the individual analyzed poplars was dominated by (*E*)-*p*-cinnamyl coumarate in *P. balsamifera* (7.74% TIC), caffeic acid phenethyl ester (CAPE) in *P. × berolinensis* (1.39% TIC), and 3-methyl-2-butenyl (*E*)-caffeate in *P. × canadensis* ‘Marilandica’ (2.46% TIC). However, only traces of the latter compound were found in *P. wilsonii* buds (Table 1).

Among the studied poplars, phenylpropanoids made the largest contribution to the chemical composition of *P. balsamifera* buds. In the set of these compounds, eight new ones, not described for this species, were discovered, namely n-propyl-, isobutyl-, isopentyl, 3-methyl-3-butenyl-, 2-methyl-2-butenyl *p*-coumarates, isopentyl-, 3-methylbutanyl-, and cinnamyl caffeates. In the buds of *P. × berolinensis* and *P.* × *canadensis* ‘Marilandica’, the presence of benzyl-, 3-methyl-3-butenyl caffeates, and 2-methyl-2-butenyl *p*-coumarate was found. Moreover, in the buds of *P. × berolinensis* isopentyl-, 2-methyl-2-butenyl, and hydrocinnamyl caffeates were identified, and 3-methyl-2-butenyl ferulate and 2-phenylethyl *p*-coumarate in *P.* × *canadensis* ‘Marilandica’ buds.

The promising therapeutic potential of 4-methoxycinnamyl *p*-coumarate in the treatment of neuroinflammation-related diseases has been demonstrated in vitro using murine microglial cells (BV2). This compound significantly increased the expression of heme oxygenase-1 (HO-1) by upregulating the nuclear factor erythroid 2-related factor 2 (Nrf2) pathway and suppressed the activation of NF-κB, MAPK, and Akt/GSK-3β [41].

CAPE is the most frequently studied compound for the assessment of biological activity among those identified in the *Populus* genus. So far, this compound has been reported to have antioxidant, anti-inflammatory, immunomodulatory, antiviral, antiproliferative, anti-cancer, chemopreventive, and wound-healing properties [42]. A number of studies have shown that the anti-inflammatory effect of CAPE is due to the reduction in ROS levels and the inhibition of the suppression of myeloperoxidase activity by NF-κB and the production of pro-inflammatory cytokines [42,43,44]. Both in vitro and in vivo studies have shown that CAPE specifically targets genes involved in cell death, angiogenesis, cell cycle regulation, and metastasis. CAPE can reduce the proliferation of human cancer cells through the NF-κB-signaling pathway [43].

A similar composition in the buds of *P. balsamifera*, *P. × berolinensis*, and *P.* × *canadensis* ‘Marilandica’ of various groups of compounds with anti-inflammatory activity, demonstrated in the literature, makes these raw plant materials an interesting research model for comparing and assessing the anti-inflammatory effects of poplars in the context of disclosed quantitative differences in their chemical composition (Figure 1). The planned in vitro studies using cell lines will include the assessment of the impact of individual extracts on the activity of cyclooxygenase 1 and 2, and the gene expression of proteins involved in the inflammatory process, IL-1β, IL-6, IL-8, IL-10, TNF-α, etc.

One of the lesser-known groups of compounds found in leaf buds of the *Populus* genus is sesquiterpenes. So far, their occurrence has been demonstrated only in three poplar taxa, *P. nigra*, *P. balsamifera*, and *P. × canadensis* [16,23,28]. Against the background of these rare literature data, the obtained results of GC-MS analyses expand the knowledge of the presence of sesquiterpenes in the buds of *P. × berolinensis* and *P. wilsonii*. The analyzed *P. × berolinensis* buds were characterized by a high concentration of sesquiterpenes (23.68% TIC) with the dominance of hinesol (9.0% TIC) and β-eudesmol (5.99% TIC). The remaining buds tested were characterized by a much lower content of these compounds. In this group of compounds, γ-curcumene dominated in the buds of *P. balsamifera*, whereas α-cadinol dominated in the plant material of *P.* × *canadensis* ‘Marilandica’. On the other hand, in the examined *P. wilsonii*, sesquiterpenes occurred only in trace amounts (Table 1).

The previously analyzed essential oils from the buds of *P. nigra* and *P.* × *canadensis* were characterized by a high eudesmol content, whereas hexane extracts of *P. balsamifera* buds were dominated by guaiol [16,23,40]. The presence of cadinol, δ- and γ-cadinenes, β- and γ-eudesmols, α-muurolene, and α-calacorene was confirmed in the buds of Portuguese *P.* × *canadensis* [16].

The leaf buds of *P. × berolinensis* are, compared to the other examined, an interesting plant material for assessing the impact of a high content of sesquiterpenes, in addition to a significant share of flavonoids and phenylpropanoids, on the biological activity of poplars.

The examined buds of *P. wilsonii* were distinguished by the presence of phenylpropenoid glycerides, among which 2-acetyl-1-*p*-coumaroyl-3-caffeoylglycerol (lasiocarpin B), 2-acetyl-1-caffeoyl-3-feruloylglycerol and 1, 3-di-*p*-coumaroyl-2-acetylglycerol (lasiocarpin A) were identified as the dominant ones (Table 1). Two of them, lasiocarpin A and B, were isolated from the buds of another representative of the Leucoides section of the *Populus* genus, namely *P. lasiocarpa* [45]. This indicates that glycerol esters are compounds characteristic of the species of this section of poplar. Moreover, the presence of these compounds has so far been found in the buds of *P. tremula* from the Leuce section, *P. szechuanica* from Tacamahaca, and *P. euphratica* from Turanga [19,24,46,47].

The studied poplar buds constitute a valuable material for the isolation of a number of interesting chemical compounds from the group of phenylpropenoids and their glycerides, which are not commercially available.

The diversity of the chemical composition of *Populus* buds makes them an interesting plant material for testing the impact of the presence or absence of a given group of compounds on the biological properties and potential synergistic effects. Taking into account the traditional use of poplar buds and the demonstrated presence of compounds with anti-inflammatory and antibacterial effects proven in the literature, the buds of *P. balsamifera* and *P. × berolinensis* should be selected from those examined as material for further in vivo studies to assess their effectiveness in the treatment of bacterial dermatitis. The detailed identification of the chemical composition of analyzed poplar buds is important for the interpretation of future planned research on their biological activity.

### 3.2. 2D-HPTLC Analysis

As mentioned in the introduction, a basic and very important element of the standardization of a medicinal raw plant material (herbal medicine) is its correct botanical and phytochemical identification. For this purpose, in addition to morphological and anatomical analysis, chromatographic methods, including TLC, are used. Taking into account the complex chemical composition of poplar buds revealed and described above, comprising numerous compounds from the phenolic group (alcohols, phenolic acids, and flavonoids) and various substances such as terpenes, sesquiterpenes, aliphatic acids, and glycerol derivatives (Table 1), the technique 2D-TLC (two-dimensional thin-layer chromatography) was used.

The developed method enabled the separation of approximately 30–35 compounds, depending on the examined poplar variety, whereas in the 1D-TLC systems used, only 16 compounds were separated (unpublished data). Optimization experiments (Figure 2) were performed based on 2D-TLC separation conditions previously used in the quantitative analysis of flavonoids and phenolic acids in propolis samples [48] and the results of our previous work on TLC separation of flavonoid aglycones in extracts from poplar buds [20]. Balsam poplar bud extract was used for this study, which, according to the results of GC-MS analyses, was characterized by a very rich and diverse chemical composition.

In the next stage, the suitability of various detection reagents indicated in the literature as characteristic for phenolic acids and flavonoids was tested [49,50,51]. Chromatograms developed using the spraying reagents were analyzed at daylight and UV λ—366 nm. The most effective in the analysis of poplar bud extracts were 2% methanol solution of AlCl_3_ and 0.5% solution of anisaldehyde in 5% methanol solution of sulfuric acid (Table 2). It should be noted that the use of a reagent-containing anisaldehyde requires activation of the HPTLC plate (washing with chloroform and then drying at 100 °C for 30 min) in order to remove factory impurities of the adsorbent. Otherwise, as a result of developing the chromatogram in a given mobile phase, they accumulate in its upper right corner and, as a result of the derivatization process, turn purple, covering the spots of compounds migrating in this part, including pinostrobin (Figure 2E and Figure 3F).

Greater variation in the color of spots of individual compounds within groups was observed when using a detection reagent containing anisaldehyde than when using aluminum chloride. Additionally, the obtained chromatograms were observed in visible and ultraviolet light at λ—366 nm, which extended the possibilities of identifying a given compound due to its different color in visible light and fluorescence at λ—366 nm (Figure 3E,F, Table 2). In visible light, spots of phenolic acids, including cinnamic acid derivatives, appear as greyish-pink, among which *p*-coumaric acid stands out with an intense pink color. This is a very valuable property when identifying this compound in TLC chromatograms because it does not give selective color reactions with other reagents used. Most spots of flavonoid compounds are yellow in color, whereas hydroxyl derivatives of flavanone (pinocembrin, naringenin, eriodictyol) and flavanonol (pinobanksin) are orange in color. However, the methoxy derivative of pinocembrin—pinostrobin and its chalcone—were violet in color (Figure 3F). In contrast, under ultraviolet light, after derivatization with anisaldehyde, flavanones exhibit brownish-red fluorescent zones, clearly distinguishing them from the greenish-yellow or light blue fluorescent zones of flavones and flavonols (Figure 3E, Table 2).

On the other hand, the use of a 2% AlCl_3_ and the detection of compounds under UV light with a wavelength of λ—366 nm results in an intense blue fluorescent zone of caffeic acid and differentiates the fluorescence of galangin and chrysin—light blue and greenish-yellow, respectively (Figure 3B,C, Table 2). When using a spraying reagent with anisaldehyde, the fluorescence of this phenolic acid is less intense and similar to other compounds.

The optimized 2D-HPTLC method enabled the separation of methanol extracts from the analyzed poplar buds, which in most cases are multi-component mixtures (Figure 4, Figure 5, Figure 6 and Figure 7). Among the standards used, the separation of chrysin and galangin as well as *p*-coumaric acid from caffeic acid phenethyl ester was achieved, unlike the 1D-HPTLC method (Figure 3C,F). Moreover, it was noted that the spot identified as pinocembrin, as a result of the previously performed 1D-HPTLC separation of the extract from *P. balsamifera* buds, is a mixture of two compounds (Figure 4).

The developed 2D-HPTLC method is a fast, simple, and relatively cheap method for assessing the botanical identity of poplar buds, which involves identifying characteristic compounds present in a given *Populus* species or variety. At the same time, in the tested poplar buds, it enables bioautographic visualization of compounds showing antioxidant activity using ABTS and DPPH solutions (Figure 4E,J, Figure 5E,J, Figure 6E,J and Figure 7E,J).

The buds of *P. balsamifera*, *P. × berolinensis*, and *P. × canadensis* ‘Marilandica’ contained a number of compounds with the ability to scavenge free radicals, which belonged primarily to the group of simple phenols and polyphenols. In turn, ABTS and DPPH bioautograms of buds of *P. wilsonii*, a species rich in glycerol derivatives, showed only the presence of a few compounds with antioxidant properties.

### 3.3. Antioxidant Capacity

The antioxidant activity of the tested buds of *Populus* ranged from 2.41 to 7.57 mM TEA/g d.w. in the DPPH assay, 3.65 to 12.10 mM TEA/g d.w. in ABTS, and 13.39 to 24.68 mM TEA/g d.w. in the FRAP test. Statistically significant differences in the antioxidant capacity were found between all tested bud extracts in the DPPH and ABTS tests, whereas in the FRAP test, only the extract from *P. × berolinensis* differed from the others (Table 3). However, what is interesting and requires explanation in further research is the fact that in the FRAP test, no statistically significant differences were found between extracts from the buds of *P. wilsonii*, *P. balsamifera*, and *P.* × *canadensis* ‘Marilandica’ (Table 3).

### 3.4. Total Flavonoid (TFC) and Total Phenolic (TPC) Content

Quantitative analysis of flavonoids (TFC) in the tested poplar buds confirms the preliminary results of analyses using 2D-HPTLC and GC-MS methods. Extracts from *P. balsamifera*, *P. × canadensis* ‘Marilandica’, and *P. × berolinensis* contained 44.76, 55.17, and 65.01 mg/g RE, respectively, whereas *Populus wilsonii* contained only 7.92 mg/g RE. Unlike other taxa, only a few flavonoids were found in *P. wilsonii* by chromatographic methods (Table 1, Figure 7).

The determined content of flavonoid compounds in the buds of *Populus balsamifera* (44.76 mg/g) was very similar to the content in raw plant material from Lithuania (39.33–45.26 mg/g) [13,14].

Significant differences were also found in the TPC of the analyzed poplar buds. The extract of *P. wilsonii* contained 64.96 mg/g GAE, whereas in the remaining taxa tested, TPC ranged from 141.34 to 183.113 mg/g GAE (Table 3).

The determined TPC is similar to the TPC in the buds of *P. nigra* from Romania (106.57 mg/g GAE [12]), and *P. balsamifera* and *P. nigra* buds (196.81 mg/g and 95.02 mg/g) from Lithuania (expressed in *p*-coumaric acid equivalents) [14]. In the buds of *Populus* × *berolinensis*, *P. × canadensis* ‘Marilandica’, and *P. wilsonii*, both TFC and TPC were determined for the first time.

The conducted research has shown that the leaf buds of *P. × berolinensis* are characterized by the most diverse chemical composition, the highest content of flavonoids and phenols, and the highest antioxidant capacity, which allows us to assume that they may constitute a potential herbal medicine with anti-inflammatory effects. However, further in vitro and in vivo studies using different inflammation models are needed to verify this hypothesis.

## 4. Materials and Methods

### 4.1. Chemicals

All solvents were of analytical grade. Ethyl acetate, methanol, chloroform, diethyl ether, sodium hydroxide (NaOH), ammonium hydroxide 25%, sulphuric acid (VI) 95% (H_2_SO_4_), formic acid 98–100%, Folin and Ciocalteu’s phenol reagent, sodium nitrate, sulfanilic acid, and vanillin were purchased from POCH (Gliwice, Poland); hexane, acetone, and methanol for spectroscopy, Uvasol, hydrochloric acid (HCl) fuming 37%, and magnesium acetate tetrahydrate were from Merck (Darmstadt, Germany); and ethanol 95% was from Polmos (Starogard, Poland). Aluminum chloride hexahydrate (AlfaAesar, Kandel, Germany), sodium carbonate anhydrous (Chempure, Piekary Śląskie, Poland), and ethyl methyl ketone (2-butanon) were from Chem-Lab NV (Zedelgem, Belgium), acetic acid 99.8%, sodium acetate, sodium persulfate, 2-aminoethyl diphenylborinate (Natural Product Reagent—NPR), Kollisolv PEG E 400 (Polyethylene glycol 400, Macrogol 400), p-anisaldehyde, 2,2-diphenyl-1-picrylhydrazyl (DPPH), 2,2′-azino-bis(3-ethylbenzothiazoline-6-sulfonic acid) diammonium salt (ABTS), 2,4,6-Tris(2-pyridyl)-s-triazine (TPTZ), iron(III) chloride hexahydrate (FeCl_3_ × 6H_2_O), (±)-6-hydroxy-2,5,7,8-tetramethylchromane-2-carboxylic acid (Trolox), pyridine, and bis(trimethylsilyl)trifluoroacetamide (BSTFA) containing 1% trimethylchlorosilane were purchased from Sigma-Aldrich (Steinheim/Darmstadt, Germany).

Demineralized water was prepared using a Merck Millipore Water Purification System, Millipore (Molsheim, France).

Galangin, pinocembrin, pinostrobin, rutin, and picein were purchased from Extrasynthèse (Genay, France), caffeic acid and kaempferol from Fluka (Buchs, Switzerland), chrysin, pinobanksin, salicin, and gallic acid from Sigma-Aldrich (Steinheim, Germany), and *p*-coumaric acid from Koch-Light (Colnbrook, UK). The standards were dissolved in methanol (1 mg/2 mL).

### 4.2. Plant Material

Leaf buds of two species and two hybrids from genus *Populus*, *P. balsamifera* L. (Bl), *P. × berolinensis* Dippel (K.Koch) (Br), *P. × canadensis* Moench ‘Marilandica’ (syn. *P. × canadensis* var. *marilandica* Rehder (Rehder)) (Mr), and *P. wilsonii* C.K.Schneid. (Ws), were collected in March, 2016 from trees growing in Gdańsk (Poland) and then dried. Plant material was botanically classified by Mrs. Jolanta Zarembska, the taxonomist of the Medicinal Plants Garden of the Medical University of Gdańsk. The voucher specimens of these plant materials (16B-015, 16B-016, 16B-014, 16B-019) were deposited at the Department of Pharmacognosy, Medical University of Gdańsk.

### 4.3. Sample Preparation

#### 4.3.1. Methanol Extracts

Dried buds of poplars (1 g) were manually crushed and then extracted three times with methanol (30 mL, 60 °C) on a magnetic stirrer (45 min). The obtained extracts were combined and evaporated to dryness and then dissolved in methanol (15 mL).

#### 4.3.2. Ether Extracts

Dried buds of poplars (2 g) were manually crushed and then extracted with diethyl ether (15 mL) by shaking (60 s). The obtained extract was filtered with a paper filter and the solvent was evaporated to dryness.

### 4.4. GC-MS Analysis

#### 4.4.1. Preparation of TMS Derivatives and Their GC-MS Analysis

For the preparation of TMS derivatives, 5−10 mg of the ether bud extract was put into a vial of 2 mL in volume and dissolved in 240 μL of pyridine and then 60 μL of BSTFA was added. The reaction mixture was heated for 0.5 h at 60 °C to form trimethylsilyl (TMS) derivatives.

The solutions of TMS derivatives were analyzed by GC−MS on an HP 7890A gas chromatograph with a 5975C VLMSD mass selective detector (MSD) with a Triple-Axis Detector (Agilent Technologies, Santa Clara, CA, USA). The apparatus was fitted with an HP-5MS capillary column (30 m × 0.25 mm i.d., 0.25 μm film thickness) with an electronic pressure control and a split/splitless injector. The latter worked at 220 °C in the split (1:50) mode. The helium flow rate through a column was 1 mL/min in a constant flow mode. The sample volume was 1.0 μL. The initial column temperature was 50 °C, rising at 3 °C/min to 320 °C. To identify the components, both mass spectral data and the calculated retention indices were used. The MSD acquisition parameters were as follows: transfer line temperature was 280 °C, MS source temperature was 230 °C, and MS Quard temperature was 150 °C. The EI mass spectra were obtained at an ionization energy of 70 ev. The MSD was set to scan 41–600 a.m.u. After integration, the fraction of each component in the total ion current (TIC) was calculated. The described procedure was carried out in triplicate.

#### 4.4.2. Retention Indices Determination and Component Identification

Linear temperature programmed retention indices (*I*^T^) were calculated from the results of the separation of the hexane solution of the C_8_–C_40_ *n*-alkanes calibration standard and prepared samples of derivatized extracts. At present, the *n*-alkanes calibration standards are commercially available. However, high-temperature capillary chromatography allows for the separation of high-boiling compounds with *I*^T^ values higher than 4000. To calculate the retention indices for such components, one needs the corresponding reference compounds, *n*-alkane homologs with *n* > 40. For this purpose, we extracted (using *n*-hexane) Parafilm M^®^ foil containing small amounts of higher *n*-alkanes [52]. The retention times of linear C_40_–C_45_ homologs were registered as the single ion (*m*/*z* 57).

The components were identified using an automatic data-processing system included with the equipment used. Mass spectrometric identification was carried out with an automatic system of GC–MS data processing supplied by NIST mass spectra library and the recently published database of retention indices and mass spectra of TMS derivatives [52]. The latter contains more than 1800 spectra and *I^T^* values of TMS derivatives prepared from authentic preparations of flavonoids and other phenolics, as well as terpenoids, aliphatic acids, alcohols, and carbohydrates.

The hexane solution of C_10_–C_40_ *n*-alkanes was separated under the above conditions. The linear temperature-programmed retention indices of the registered components were calculated from the results of the separation of this solution and silanized bud extracts and were compared with the collections [52,53]. The identification was considered reliable if the results of the computerized search of the mass spectra library were confirmed by the experimental *I*^T^ values, i.e., if their deviation from the averaged published index value did not exceed ±10 u.i. In the case of a greater deviation of the *I*^T^ values from those given in databases [52,53], or in the absence of the literature values for this independent parameter, the identification is considered tentative and the name of the compound is marked with a question mark.

### 4.5. 1D-(One) and 2D-HPTLC (Two-Dimensional High-Performance Thin-Layer Chromatography)

The 1D- and 2D-HPTLC experiments were performed on 10 cm × 10 cm TLC Si 60 F_254_ plates (Merck, Darmstadt, Germany), which were activated by washing with chloroform and drying at 100 °C for 30 min. The mobile phase chloroform:methanol:formic acid (95:2:3 *v*/*v*/*v*) was used in the first dimension (1D) and n-hexane:ethyl acetate:formic acid (60:40:1.3 *v*/*v*/*v*) in the second (2D). The methanolic extracts of poplar buds (10 µL) were applied as 6 mm bands (1D) or 5 mm spots (2D) to the HPTLC plates with a semi-automatic TLC sampler AS-30 (Desaga, Darmstadt, Germany) and developed in a saturated (10 min) Twin Trough Chamber (Camag, Muttenz, Switzerland) to a distance of 7 cm at room temperature.

The suitability of various detection reagents indicated in the literature as characteristic of phenolic acids and flavonoids was tested. These were a 2% methanolic solution of aluminum chloride (III) (2% AlCl_3_), 1% methanolic solution of magnesium acetate, ammonium hydroxide 25% vapors (NH_3_), diazotized sulfanilic acid, 1% ethanolic solution of vanillin:hydrochloric acid 37% (100:3, *v*/*v*) [51], 1% ethanolic solution of 2-aminoethyl diphenylborinate NPR (10 g/L) and the solution of Macrogol 400 (PEG) (50 g/L) in MeOH [50], and 0.5% solution of anisaldehyde in 5% sulfuric acid in methanol (95 °C, 3 min) [49]. Chromatograms developed using the above reagents were analyzed in daylight, with UV λ—366 nm.

As a result of the above experiments, developed and dried plates were analyzed in daylight (λ—500 nm) and UV light at λ—366 nm, before and after the derivatization by 2% methanolic solution of AlCl_3_ or 0.5% solution of anisaldehyde in 5% sulfuric acid in methanol (95 °C, 3 min). Spraying reagents were spread on developed and dried plates using an automated spraying device—Derivatizer (Camag, Muttenz, Switzerland). Documentation of chromatograms was performed using the TLC Visualizer (Camag, Muttenz, Switzerland).

### 4.6. Bioautography Tests

#### 4.6.1. DPPH Test

Developed and dried HPTLC plates were dipped in the 0.05% methanolic solution of DPPH with the use of a Chromatogram Immersion Device (Camag, Muttenz, Switzerland). Then, the plates were stored for 30 min in a dark place and analyzed in daylight. Compounds with scavenging properties of the DPPH free radical were visible as yellow spots on the purple background of the chromatograms [54].

#### 4.6.2. ABTS Test

A 7 mM solution of ABTS was created by dissolving 38.41 mg of ABTS into 8 mL of demineralized water. A 2.45 mM solution of sodium persulfate (K_2_S_2_O_8_) was made by dissolving 66.2 mg of K_2_S_2_O_8_ into 10 mL of demineralized water. Then, 1 mL of 2.45 mM K₂S₂O₈ was added to 7 mM ABTS. The obtained mixture was filled up to 10 mL with water and left in the refrigerator (4 °C) for 16 h. After this time, developed and dried HPTLC plates were sprayed with the use of an automated spraying device, the Camag Derivatizer. Next, the plates were stored for 6 min in a dark place and analyzed in daylight. Bright spots of compounds with antiradical properties were observed on the blue background of the chromatograms [55].

The documentation of chromatograms and bioautograms was performed using the TLC Visualizer (Camag, Muttenz, Switzerland).

### 4.7. Antioxidant Capacity Assays

Results of all the tests described below are expressed as mM Trolox equivalents per g of the dry weight of buds (mM TE/g d.w.).

#### 4.7.1. DPPH Assay

The DPPH assay was performed using a method previously described by Tuberoso et al. [56] with some modifications. The freshly prepared 0.04 mM solution of DPPH in methanol was subjected to ultrasound (5 min) and then placed in the refrigerator (4 °C) for 60 min. The standard curve was linear between 0.02 and 0.1 mM Trolox.

Then, 350 μL of the plant extracts or Trolox dilution were mixed with 2.5 mL of 0.04 mM DPPH solution in brown glass bottles and stored in a dark place for 30 min. The blank sample consisted of 350 μL of methanol and 2.5 mL of a 0.04 mM DPPH solution. Spectrophotometric measurements were made at λ—517 nm.

#### 4.7.2. FRAP Assay

The FRAP assay was carried out using a method previously described by Benzie and Strain [57] with some modifications. To perform the FRAP test, the following reagents were made: a 300 mM acetate buffer (pH 3.6), a 10 mM solution of TPTZ in 40 mM HCl, and 20 mM of FeCl_3_ × 6H_2_O. The prepared solutions were mixed in a ratio of 10:1:1 (*v*/*v*/*v*) and then heated to 37 °C (3 min) in a water bath. The standard curve was linear between 0.02 and 0.48 mM Trolox.

For testing, 150 μL of the analyzed extracts or Trolox dilution were mixed with 3 mL of previously prepared FRAP reagent. Samples were incubated in the dark for 30 min. The blank sample consisted of 150 μL of water and 3 mL of FRAP reagent. Absorbance was measured at λ—593 nm.

#### 4.7.3. ABTS Assay

The ABTS assay was performed using a method previously described by Thaipong et al. [58], with some modifications. For this test, 2 mL of 7 mM ABTS solution was mixed with 0.35 mL of 140 mM sodium persulfate and the prepared mixture was incubated for 15 h in a refrigerator (4 °C) with no access to light. After this time, the reagent was diluted with water in a ratio of 1:90 (*v*/*v*) while maintaining a constant absorbance, i.e., 0.7 ± 0.02. The standard curve was linear between 0.02 and 0.12 mM Trolox.

For testing, 200 μL of the analyzed extracts or appropriate Trolox dilution were taken and 2 mL of previously prepared ABTS reagent was added. The blank sample consisted of 200 μL of water and 2 mL of ABTS reagent. The absorbance was measured after 6 min at λ—734 nm.

### 4.8. Total Phenolic Content (TPC)

The TPC was estimated according to the Folin–Ciocalteu method [59] and was expressed as mg of gallic acid equivalent (GAE) per g of dry weight (d.w.) in relation to the calibration curve of GA (y = 1.2405x + 0.0250; r^2^ = 0.9996) in a concentration range of 0.2–1.0 mg/mL.

### 4.9. Total Flavonoid Content (TFC)

The TFC was determined using the method described by Barman et al. [60] and was expressed as mg of rutin equivalent (RE) per g of d.w. in relation to the calibration curve (y = 0.0034x + 0.0096; r^2^ = 0.9997) in a concentration range of 6.4–102.4 mg/mL.

Absorbance in all the above tests was measured by spectrophotometer UV-1800 (Shimadzu, Kyoto, Japan).

### 4.10. Statistical Analysis

The mean difference between the TFC, TPC, and the DPPH, ABTS, and FRAP antioxidant capacity was controlled using the one-way analysis of variance (ANOVA) followed by Tukey’s multiple comparison tests. All statistical analyses were performed using Statistica 12 (StatSoft, Kraków, Poland).

## 5. Conclusions

The research carried out showed significant differences in the chemical composition (GC-MS identification, TPC and TFC determination) and antioxidant capacity (spectrophotometric DPPH, ABTS, and FRAP assays) of the buds of the studied *Populus* taxa. The composition of *P. wilsonii* buds was analyzed for the first time and the experiments performed provided new information about the types of biologically active compounds found in buds of *P. balsamifera*, *P. × berolinensis*, and *P. × canadensis* ‘Marilandica’.

The 2D-HPTLC method for metabolomic profiling of the studied poplars was developed and could be used in a pharmacopoeial monograph of *Populi gemmae* for their botanical identification. This method can also be used to assess the botanical origin of propolis.

The plant material from *P. × berolinensis* had the richest chemical composition and the highest content of flavonoids and phenols, which determines the highest antioxidant capacity. In our previous studies [9], the buds of this poplar hybrid showed the strongest anti-inflammatory effect, and the results obtained confirm the influence of their rich chemical composition on biological activity. The above data indicate that *P. × berolinensis* buds may be a potential herbal medicine with valuable anti-inflammatory effects. Further research will be conducted to explain the mechanisms of action of this plant material at the molecular level.

## Figures and Tables

**Table 1:** Chemical composition (% of TIC) of ether extracts from the leaf buds of *Populus balsamifera* (Bl), *P. × berolinensis* (Br), *P. × canadensis* ‘Marilandica’ (Mr), and *P. wilsonii* (Ws).

Compound	RI^Exp^	RI^DB^	TIC [%]			
			Bl	Br	Mr	Ws
**Sesquiterpene and sesquiterpenoids**						
γ-Curcumene	1478	1480	0.37	- *	-	-
ar-Curcumene	1482	1883	0.24	-	trace **	-
α-Muurolene	1500	1499	-	0.56	trace	-
Sesquicineol, TMS ***	1508	1514	0.19	-	-	-
δ-Cadinene	1522	1524	-	-	0.23	-
α-Calacorene	1543	1544	-	0.23	trace	-
α-Copaene-11-ol, TMS	1628	1630	-	0.72	-	-
1-*epi*-Cubenol, TMS	1662	1665	-	0.58	-	-
Cubenol, TMS	1677	1672	-	1.96	-	-
Acorenol, TMS	1723	1722	-	1.88	trace	-
Agaraspirol, TMS	1734	1733	-	0.58	-	-
Hinesol, TMS	1736	1736	-	9.00	-	-
γ-Eudesmol, TMS	1743	1741	-	-	0.22	trace
α-Cadinol, TMS	1746	1747	-	-	0.28	-
β-Eudesmol, TMS	1749	1750	-	5.99	0.17	trace
**Phenolic acids**						
Benzoic acid, TMS	1248	1248	0.29	trace	trace	0.96
4-Methoxycinnamic acid, TMS	1828	1830	trace	0.55	-	-
*p*-Coumaric acid, di-TMS	1943	1947	15.36	1.15	0.88	0.93
Isoferulic acid, di-TMS	2087	2089	-	1.95	0.72	-
(*E*)-Ferulic acid, di-TMS	2100	2103	-	trace	0.52	0.15
3,4-Dimethoxycinnamic acid, TMS	2033	2035	-	3.65	trace	-
Caffeic acid, tri-TMS	2154	2154	1.17	4.58	4.92	2.49
**Benzoic acid derivatives**						
Isoamyl benzoate	1432	1430	0.26	-	-	-
Benzyl benzoate	1753	1560	0.36	-	-	-
2-Phenylethyl benzoate	1844	1844	0.53	-	-	-
Benzyl salicylate, TMS	2022	2027	1.89	-	-	-
**Phenylpropenoids (cinnamic acid derivatives)**						
*n*-Propyl *p*-coumarate, TMS	1975	1975	0.29	-	-	-
Isobutyl *p*-coumarate, TMS	2026	2030	1.31	-	-	-
Isopentyl *p*-coumarate, TMS	2143	2147	4.31	-	-	-
3-Methyl-3-butenyl *p*-coumarate, TMS	2151	2151	0.39	-	-	-
2-Methyl-2-butenyl (*E*)-*p*-coumarate, TMS	2200	2204	4.68	trace	0.19	-
Isopentyl (*E*)-caffeate, di-TMS	2360	2359	0.36	0.30	-	-
2-Methylbutanyl (*E*)-caffeate, di-TMS	2356	2355	0.29	-	-	-
3-Methyl-3-butenyl (*E*)-caffeate, di-TMS	2368	2369	-	0.80	0.98	-
3-Methyl-2-butenyl (*E*)-ferulate, TMS	2375	2374	-	-	0.70	-
2-Methyl-2-butenyl (*E*)-caffeate, di-TMS	2413	2411	-	1.11	-	-
3-Methyl-2-butenyl (*E*)-caffeate, di-TMS	2423	2325	-	0.80	2.46	trace
2-Phenylethyl *p*-coumarate, di-TMS	2600	2601	1.88	-	0.15	-
Benzyl (*E*)-caffeate, di-TMS	2716	2723	-	0.35	0.36	-
Caffeic acid phenethyl ester (CAPE), di-TMS	2799	2805	0.21	1.39	1.55	-
Cinnamyl (*E*)-*p*-coumarate, TMS	2824	2835	7.74	-	-	-
Hydrocinnamyl caffeate, di-TMS	2922	2927	-	0.20	-	-
Cinnamyl (*E*)-caffeate, di-TMS	3035	3040	0.45	-	-	-
**Phenylpropenoid glycerides**						
1-Acetyl-3-*p*-coumaroylglycerol, di-TMS	2580	2580	-	-	-	0.27
1-Acetyl-3-*p*-caffeoylglycerol, di-TMS	2763	2763	-	-	-	0.27
1,3-di-*p*-Coumaroylglycerol, tri-TMS	3865	3870	-	-	-	1.19
1,3-di-*p*-Coumaroyl-2-acetylglycerol, di-TMS	3955	3964	-	-	-	16.73
1-Caffeoyl-3-*p*-coumaroylglycerol, tetra-TMS	4020	4026	-	-	-	1.00
2-Acetyl-1-*p*-coumaroyl-3-feruloylglycerol, di-TMS	4136	4139	-	-	-	0.30
2-Acetyl-1-*p*-coumaroyl-3-caffeoylglycerol, tri-TMS	4168	4170	-	-	-	27.16
2-Acetyl-1-caffeoyl-3-feruloylglycerol, tri-TMS	4211	4209	-	-	-	25.87
**Flavonoids**						
Pinostrobin, TMS	2500	2506	6.64	6.85	1.05	-
Pinocembrin, di-TMS	2552	2548	3.94	13.18	7.86	0.13
Pinobanksin, tri-TMS	2608	2610	1.00	1.31	12.55	0.10
Pinobanksin 3-acetate, di-TMS	2690	2693	2.24	4.12	11.78	0.11
Chrysin, di-TMS	2740	2747	-	2.46	12.38	0.10
Galangin, tri-TMS	2764	2768	2.61	3.58	18.59	0.53
Pinobanksin 3-isobutanoate, di-TMS	2791	2795	-	-	1.18	-
Isosakuranetin, di-TMS	2812	2817	3.01	0.61	-	-
2′,6′,4-Tihydroxy-4-methoxyflavone, tri-TMS	2820	2821	-	-	0.43	-
Pinobanksin 3-*n*-butanoate, di-TMS	2849	2849	-	-	0.33	-
Sakuranetin, di-TMS	2877	2877	0.92	0.87	0.10	-
Pinobanksin 3-pentanoate, di-TMS	2884	2885	-	-	1.26	-
Pinobanksin 5-pentenoate, di-TMS	2964	2962	-	-	0.24	-
Pinobanksin 3-hexanoate, di-TMS	3032	3035	-	-	0.22	-
Acacetin, di-TMS	3049	3066	0.69	-	-	-
Kaempherol, tri-TMS	3109	3114	0.42	0.20	0.95	-
Apigenin, tri-TMS	3158	3159	-	0.21	1.49	-
Rhamnetine, tetra-TMS	3234	3233	-	-	0.24	-
Isorhamnetine, tetra-TMS	3245	3243	-	-	0.23	-
3,4′-Dimethyl quercetine, tri-TMS	3266	3262	-	-	0.29	-
3-Methylquercetin, tetra-TMS	3291	3292	-	-	0.33	-
Pinobanksin 3-hydrocinnamate, tri-TMS	3452	3449	-	-	0.55	-
Catechin, penta-TMS	2930	2936	-	-	-	0.31
**Chalcones**						
2′,6′-Dihydroxy-4′-methoxydihydrochalcone, di-TMS	2416	2417	5.52	-	trace	-
2′,4′,6-Trihydroxydihydro-chalcone, tri-TMS	2456	2458	2.51	0.57	trace	-
Pinostrobin chalcone, di-TMS	2504	2507	3.66	0.87	trace	-
2′,6′,α-Trihydroxy-4′-methoxychalcone, tri-TMS	2602	2601	-	trace	0.63	-
2′,6′-Dihydroxy-4,4′-dimethoxydihydrochalcone, di-TMS	2650	2655	0.57	-	-	-
2′,4′,6′-Trihydroxy-4-methoxydihydrochalcone, tri-TMS	2692	2690	2.24	-	-	-
**Aliphatic acids**						
Hexadecanoic (palmitic) acid, TMS	2052	2052	0.29	0.32	0.23	1.55
α-Linolenic acid, TMS	2215	2215	0.12	0.34	0.17	0.52
**Triterpenoids**						
Lanosterol, TMS	3329	3331	-	-	-	0.53
α-Amyrin, TMS	3374	3378	-	1.43	-	0.22
Dipterocarpol, TMS	3507	3509	-	-	-	4.32
**Other**						
*n*-Tricosane	2300	2300	0.25	0.25	trace	trace
Oxylipin, tri-TMS	2493	2494	-	0.16	0.71	trace
*n*-Pentacosane	2500	2500	-	-	trace	0.23
3-Hydroxyhexadecanoic acid, di-TMS	2620	2621	-	0.32	-	-
*n*-Heptacosane	2700	2700	0.21	0.41	0.21	0.71
1-Tetracosanol, TMS	2757	2754	-	0.35	-	-
Tetracosyl acetate	2814	2815	-	0.22	-	-
1-Hexacosanol, TMS	2954	2951	-	0.28	-	0.13
Sesquiterpene and sesquiterpenoids			0.80	23.68	1.00	trace
Triterpenoids			trace	6.66	1.71	11.76
Phenylpropenoids (cinnamic acid derivatives)			30.85	16.82	13.43	3.57
Phenylpropenoid glycerides			-	-	-	74.94
Flavonoids and chalcones			41.28	34.44	79.51	1.38
Aliphatic acids			0.12	1.06	1.29	3.28
Other			17.08	4.48	0.60	3.53
NN			9.87	12.86	2.46	1.53

RI—retention indices, * component not found, ** less than 0.01% TIC, *** TMS—trimethylsilyl derivative of compound, NN—component not identified.

**Table 2:** *h*Rf (R_f_ × 100) values of compounds identified in poplar buds extracts by 2D-HPTLC method and the colors of their zones in visible light and fluorescence at UV light, detected using different spraying reagents.

	Compound	*h*R_f_	y (mm)	Detection Method/Spraying Reagent/Visible Light/UV			
				Anisaldehyde UV at λ—366 nm	Anisaldehyde Visible Light at λ—500 nm	UV λ—366 nm	2%AlCl_3_ UV λ—366 nm
1	pinostrobin	94	60	brownish-red	violet	-	blue
2	pinocembrin	61	52	brownish-red	orange	yellowish	light blue
3	galangin	51	48	greenish-yellow	yellow	yellowish	light blue
4	chrysin	51	43	greenish-yellow	yellow	brownish-red	pale yellow
5	pinobanksin	45	43	brownish-red	orange	-	-
6	CAPE *	38	35	blue	greyish-pink	blue	blue
7	*p*-coumaric acid	49	33	pale pink	intense pink	-	-
8	kaempferol	23	39	light blue	-	yellowish	pale yellow
9	caffeic acid	20	26	blue	greyish-pink	intense blue	intense blue

* CAPE—phenethyl ester of caffeic acid.

**Table 3:** Total flavonoid (TFC) and phenolic (TPC) content, and antioxidant capacity of methanol extracts from poplar buds.

Species/Varieties of *Populus*	Compound Content *		Antioxidant Capacity [mM TEA/g d. w.] *		
	TFC [mg/g RE] *	TPC [mg/g GAE] *	DPPH	FRAP	ABTS
*P. balsamifera* (Bl)	44.76 ± 0.82 ^a^	166.17 ± 4.10 ^a,b^	3.68 ± 0.15 ^a^	13.97 ± 0.55 ^a^	12.10 ± 0.19 ^a^
*P. × berolinensis* (Br)	65.01 ± 9.30 ^b^	183.18 ± 13.97 ^a^	7.57 ± 0.51 ^b^	24.68 ± 0.55 ^b^	10.01 ± 0.34 ^b^
*P. × canadensis* ‘Marilandica’ (Mr)	55.17 ± 8.75 ^a,b^	141.34 ± 0.69 ^b^	4.46 ± 0.22 ^c^	13.39 ± 0.27 ^a^	5.67 ± 0.58 ^c^
*P. wilsonii* (Ws)	7.92 ± 0.50 ^d^	64.96 ± 13.21 ^d^	2.41 ± 0.10 ^d^	13.73 ± 0.44 ^a^	3.65 ± 0.76 ^d^

* Mean ± SD (standard deviation) (n = 3); values in individual columns marked with different letters indicate statistically significant differences (*p* < 0.05; Tukey’s RIR test).

## Data Availability

Data is contained within the article and Supplementary Material. Further inquiries can be directed to the corresponding author.

## Supplementary Materials

The following supporting information can be downloaded at: https://www.mdpi.com/article/10.3390/ijms25073971/s1.
