# Supplementary material for: Characterization of Secondary Metabolites of Leaf Buds from Some Species and Hybrids of Populus by Gas Chromatography Coupled with Mass Detection and Two-Dimensional High-Performance Thin-Layer Chromatography Methods with Assessment of Their Antioxidant Activity
Source: Int J Mol Sci. 2024 Apr 3;25(7):3971. doi: 10.3390/ijms25073971 (PMC11011796; doi:10.3390/ijms25073971)
Supplement: Supplementary file 1 [file ijms-25-03971-s001.zip › ijms-2906821-supplementary.pdf]

Table S1. Chemical composition (% of TIC) of ether extracts from the leaf buds of *Populus balsamifera* (**Bl**), *P.*× *berolinensis* (**Br**), *P.* × *canadensis* 'Marilandica' (**Mr**) and *P. wilsonii* (**Ws**).

**Compound**	**RI**^{Exp}	**RI**^{DB}		**TIC [%]**		
			**Bl**	**Br**	**Mr**	**Ws**
Benzyl alcohol, TMS	1153	1153	trace*	trace	trace	-**
2-Phenylethanol, TMS	1228	1228	trace	trace	0.22	-
Benzoic acid, TMS	1248	1248	0.29	trace	trace	0.96
1.2-Dihydroxycyclohexane, di-TMS, isomer 1	1263	1263	-	trace	-	-
1.2-Dihydroxycyclohexane, di-TMS, isomer 2	1276	1275	-	trace	-	-
Pyrocatechol, di-TMS	1324	1325	-	-	-	0.37
α-Terpineol, TMS	1323	1322	-	trace	-	-
Isobutyl benzoate	1326	1327	trace	-	-	-
Succinic acid, di-TMS	1326	1324	-	-	-	0.20
3-Phenylpropanol, TMS	1336	1336	trace	-	-	-
p-Anisic acid methyl ester	1341	1341	trace	-	-	-
α-Ylangene	1372	1372	-	trace	-	-
n-Butyl benzoate	1372	1376	trace	-	-	-
Methyl salicylate, TMS	1401	1397	trace	-	-	-
Hydrocinnamic acid, TMS	1419	1418	trace	trace	trace	-
Cinnamyl alcohol, TMS	1427	1430	trace	-	-	-
β-Copaene	1430	1432	trace	-	-	-
Isoamyl benzoate	1432	1430	0.26	-	-	-
γ-Curcumene	1478	1480	0.37	-	-	-
Prenyl benzoate?	1480	-	1.09	-	-	-
ar-Curcumene	1482	1883	0.24	-	trace	-
α-Muurolene	1500	1499	-	0.56	trace	-
β-Bisabolene	1504	1508	trace	-	-	-
Sesquicineol, TMS	1508	1514	0.19	-	-	-
γ-Cadinene	1517	1516	-	-	trace	-
δ-Cadinene	1522	1524	-	-	0.23	-
α-Calacorene	1543	1544	-	0.23	trace	-
Cinnamic acid	1549	1549	trace	-	trace	-
Protocatechuic aldehyde	1622	1620	-	-	-	0.10
α-Copaene-11-ol, TMS	1628	1630	-	0.72	-	-
epi-a-Cadinol, TMS	1644	1644	-	-	0.11	-
4-Hydroxybenzoic acid, di-TMS	1636	1636	-	-	-	0.12
Sesquiterpenoid $C_{15}H_{26}O_2$, di-TMS (111.83...238)	1649	--	-	0.22	-	-
1-*epi*-Cubenol, TMS	1662	1665	-	0.58	-	-
Cubenol, TMS	1677	1672	-	1.96	-	-
Syringaldehyde, TMS	1707	1705	-	-	-	0.10
Acorenol, TMS	1723	1722	-	1.88	trace	-
3.4-Dihydroxybenzyl alcohol, tri-TMS?	1727	-	-	-	-	0.20
Agaraspirol, TMS	1734	1733	-	0.58	-	-
Hinesol, TMS	1736	1736	-	9.00	-	-
γ-Eudesmol, TMS	1743	1741	-	-	0.22	trace
α-Cadinol, TMS	1746	1747	-	-	0.28	-
β-Eudesmol, TMS	1749	1750	-	5.99	0.17	trace

α-Bisabolol, TMS	1752	1750	trace	-	-	-
Sesquiterpenoid $C_{15}H_{24}O$, TMS? (131.73.202...277)	1752	-	-	0.18	-	-
Benzyl benzoate	1753	1560	0.36	-	-	-
Bulnesol, TMS?	1754	-	-	1.53	-	-
Vanillic acid, di-TMS	1777	1776	-	-	-	trace
Azelaic acid, di-TMS	1807	1808	-	-	-	0.06
4-Methoxycinnamic acid, TMS	1828	1830	trace	0.55	-	-
5-Phenylpent-2,4-dienoic acid, TMS	1841	-	-	-	0.16	-
2-Phenylethyl benzoate	1844	1844	0.53	-	-	-
NN (131>73.75.164.297)	1849	-	-	0.93	-	-
NN (131>73.202.159)	1892	-	-	0.40	-	-
NN (131>73.143.369)	1939	-	-	7.59	-	-
p-Coumaric acid, di-TMS	1943	1947	15.36	1.15	0.88	0.93
n-Propyl *p*-coumarate, TMS	1975	1975	0.29	-	-	-
Benzyl salicylate, TMS	2022	2027	1.89	-	-	-
Isobutyl *p*-coumarate, TMS	2026	2030	1.31	-	-	-
3,4-Dimethoxycinnamic acid, TMS	2033	2035	-	3.65	trace	-
Butenyl *p*-coumarate, TMS? (219.192.73.177.290)	2048	-	0.32	-	-	-
Hexadecanoic (palmitic) acid, TMS	2052	2052	0.29	0.32	0.23	1.55
n-Butyl *p*-coumarate, TMS	2077	2078	0.15	-	-	-
NN (131.73.162.252.292)	2083	-	-	1.87	-	-
Isoferulic acid, di-TMS	2087	2089	-	1.95	0.72	-
(*E*)-Ferulic acid, di-TMS	2100	2103	-	trace	0.52	0.15
Sesquiterpenol $C_{15}H_{24}O$, TMS? (73.157...277.292)	2139	-	1.11	-	-	-
Isopentyl *p*-coumarate, TMS	2143	2147	4.31	-	-	-
3-Methyl-3-butenyl *p*-coumarate, TMS	2151	2151	0.39	-	-	-
Caffeic acid, tri-TMS	2154	2154	1.17	4.58	4.92	2.49
2-Methyl-2-butenyl (E)-*p*-coumarate, TMS	2200	2204	4.68	Trace	0.19	-
Prenyl *p*-coumarate, TMS?	2204	2214	0.41	-	-	-
α-Linolenic acid, TMS	2215	2215	0.12	0.34	0.17	0.52
α-Linolenic + oleic acids	2221	-	trace	0.24	-	0.41
Oleic acid, TMS	2221	2222	-	-	0.19	-
NN (benzyl ester? m/z 91.373.73.329.267)	2224	-	0.21	-	-	-
NN (73>156,244.143.93.121)	2233	-	8.27	-	-	-
Isobutyl (*Z*)-caffeate, di-TMS	2245	2240	0.13	trace	-	-
Octadecanoic (stearic) acid, TMS	2251	2250	-	trace	trace	0.19
n-Tricosane	2300	2300	0.25	0.25	trace	trace
3-Methyl-3-butenyl (*E*)-ferulate, TMS	2319	2319	-	-	trace	-
NN (73.121.82.75.156)	2332	-	-	-	0.06	-
15-Hydroxyhexadecanoic acid, di-TMS	2333	2331	-	-	trace	-
NN	2346	-	-	0.12	-	-
Isopentyl (*E*)-caffeate, di-TMS	2360	2359	0.36	0.30	-	-
2-Methylbutanyl (*E*)-caffeate, di-TMS	2356	2355	0.29	-	-	-
3-Methyl-3-butenyl (*E*)-caffeate, di-TMS	2368	2369	-	0.80	0.98	-
3-Methyl-2-butenyl (*E*)-ferulate, TMS	2375	2374	-	-	0.70	-
Dihomo-γ- linoleic acid, mono-TMS?	2402	2394	-	-	0.12	-
2-Methyl-2-butenyl (*E*)-caffeate, di-TMS	2413	2411	-	1.11	-	-
2',6'-Dihydroxy-4'-methoxydihydrochalcone, di-TMS	2416	2417	5.52	-	trace	-
3-Methyl-2-butenyl (*E*)-caffeate, di-TMS	2423	2325	-	0.80	2.46	trace
Ricinoleic acid, di-TMS	2427	2428	-	-	trace	-

Eicosanoic acid, TMS	2450	2448	-	trace	-	0.13
2',4',6-Trihydroxydihydro-chalcone, tri-TMS	2456	2458	2.51	0.57	trace	-
NN (487.73.105.413.502)	2487	-	-	-	0.51	-
Oxylipin, tri-TMS	2493	2494	-	0.16	0.71	trace
Pinostrobin, TMS	2500	2506	6.64	6.85	1.05	-
n-Pentacosane	2500	2500	-	-	trace	0.23
1-*p*-Coumaroyl glycerol, tri-TMS	2528	2527	-	-	-	trace
Pinostrobin chalcone, di-TMS	2504	2507	3.66	0.87	trace	-
Pinocembrin, di-TMS	2552	2548	3.94	13.18	7.86	0.13
1-Docosanol, TMS	2556	2517	0.19	-	-	trace
NN (95.75.81.151)	2558	-	-	-	0.36	-
NN (303)	2571	-	-	1.44	0.82	-
NN (303.73)	2567	-	0.17	-	-	-
Alpinone, di-TMS (7-methylpinobanksin)?	2576	-	-	-	0.34	-
1-Acetyl-3-*p*-coumaroylglycerol, di-TMS	2580	2580	-	-	-	0.27
2-Phenylethyl *p*-coumarate, di-TMS	2600	2601	1.88	-	0.15	-
n-Hexacosane	2600	2600	-	-	-	trace
2',6',α-Trihydroxy-4'-methoxychalcone, tri-TMS	2602	2601	-	trace	0.63	-
Pinobanksin, tri-TMS	2608	2610	1.00	1.31	12.55	0.10
1.2-Diacetyl-3-*p*-coumaroulglycerol, TMS	2617	2618	-	-	-	trace
3-Hydroxyhexadecanoic acid, di-TMS	2620	2621	-	0.32	-	-
Docosanoic acid, TMS	2646	2645	-	trace	-	0.10
2',6'-Dihydroxy-4,4'-dimethoxydihydrochalcone, di-TMS	2650	2655	0.57	-	-	-
NN (flavonoid, TMS? 238.385.325.73.43.91)	2654	-	0.33	-	0.25	-
5,7-Dihydroxy-3-methoxyflavanone, di-TMS?	2673	-	-	-	1.82	-
Pinobanksin 3-acetate, di-TMS	2690	2693	2.24	4.12	11.78	0.11
2',4',6'-Trihydroxy-4-methoxydihydrochalcone, tri-TMS	2692	2690	2.24	-	-	-
n-Heptacosane	2700	2700	0.21	0.41	0.21	0.71
1-Caffeoylglycerol, tetra-TMS	2707	2708	-	-	-	trace
NN(benzyl ester? 236.221.219.91...354.339)	2712	-	0.41	-	-	-
Benzyl (*E*)-caffeate, di-TMS	2716	2723	-	0.35	0.36	-
Isalpinin (3.5-dihydroxy-7-methoxyflavone), di-TMS?	2730	-	2.88	-	-	-
Chrysin, di-TMS	2740	2747	-	2.46	12.38	0.10
3-Methylgalangin, di-TMS?	2752	2752	-	0.18	3.58	0.09
1-Tetracosanol, TMS	2757	2754	-	0.35	-	-
1-Acetyl-3-*p*-caffeoylglycerol, di-TMS	2763	2763	-	-	-	0.27
Galangin, tri-TMS	2764	2768	2.61	3.58	18.59	0.53
4'-Methylsakuranetin, TMS?	2775	-	0.84	-	-	-
Pinobanksin 3-isobutanoate, di-TMS	2791	2795	-	-	1.18	-
Caffeic acid phenethyl ester (CAPE), di-TMS	2799	2805	0.21	1.39	1.55	-
n-Octacosane	2800	2800	-	-	-	0.07
Isosakuranetin, di-TMS	2812	2817	3.01	0.61	-	-
Tetracosyl acetate	2814	2815	-	0.22	-	-
2′,6′,4-Tihydroxy-4-methoxyflavone, tri-TMS	2820	2821	-	-	0.43	-
3-Hydroxydocosanoic acid, di-TMS	2821	2819	-	trace	-	0.05
Trihydroxymethoxychalcone, tri-TMS? (502)	2826	-	-	0.24	0.21	-
Cinnamyl (*E*)-*p*-coumarate, TMS	2824	2835	7.74	-	-	-
Pinobanksin 3-*n*-butanoate, di-TMS	2849	2849	-	-	0.33	-
Tetracosanoic acid, TMS	2845		-	-	-	0.10
NN (222.73.369.223)	2850	2845	0.32	-	-	-

Sakuranetin, di-TMS	2877	2877	0.92	0.87	0.10	-
Pinobanksin 3-pentanoate, di-TMS	2884	2885	-	-	1.26	-
Pinobanksin x-pentanoate, di-TMS?	2886	-	-	-	0.23	-
NN (473. 73.296.488)	2889	2892	-	0.23	-	-
n-Nonacosane	2900	2900	-	trace	-	0.14
Hydrocinnamyl caffeate, di-TMS	2922	2927	-	0.20	-	-
Catechin, penta-TMS	2930	2936	-	-	-	0.31
1-Hexacosanol, TMS	2954	2951	-	0.28	-	0.13
Pinobanksin 5-pentenoate, di-TMS	2964	2962	-	-	0.24	-
Hesperetin, tri-TMS	2972	2977	-	trace	-	-
Pinobanksin 3-hexanoate, di-TMS	3032	3035	-	-	0.22	-
Cinnamyl (*E*)-caffeate, di-TMS	3035	3040	0.45	-	-	-
5,7,4'-Trihydroxy-3'-methylflavone, tri-TMS?	3042	-	1.05	-	-	-
NN (396.147.105.81)	3046	-	-	-	-	0.12
Acacetin, di-TMS	3049	3066	0.69	-	-	-
NN (453.175.75)	3050	-	-	-	-	0.07
3,5,4'-Trihydroxy-5-methoxyflavone, tri-TMS	3100	3096	trace	-	0.10	-
Kaempherol, tri-TMS	3109	3114	0.42	0.20	0.95	-
NN (73.103.341.143.515)	3128	-	-	0.69	-	-
5,7,4'-Trihydroxy-3-methoxyflavone, tri-TMS?	3138	-	-	-	0.66	-
α-Tocopherol, TMS	3148	3149	-	-	-	0.13
1-Octacosanol, TMS	3150	3148	-	-	-	0.11
Apigenin, tri-TMS	3158	3159	-	0.21	1.49	-
Triterpenoid (189.129.73.143)	3159	-	-	0.41	0.19	-
Triterpenoid (189.129.73.143)	3175	-	-	0.51	-	-
Triterpenoid (189.14.73.129.175)	3183	-	-	3.16	-	0.08
NN (589.73.159.143)	3222	-	-	0.40	-	-
Rhamnetine, tetra-TMS	3234	3233	-	-	0.24	-
Isorhamnetine, tetra-TMS	3245	3243	-	-	0.23	-
NN (193.73.577.219.456)	3317	-	0.16	-	-	-
3,4'-Dimethyl quercetine, tri-TMS	3266	3262	-	-	0.29	-
Triterpenoid (131)	3278	-	-	0.61	-	-
3',4',7-Trimethylquercetine	3284	3285	-	-	0.08	-
3-Methylquercetin, tetra-TMS	3291	3292	-	-	0.33	-
β-Amyrone	3307	3312	-	-	-	0.05
Sterol, TMS?	3314	-	-	-	0.19	1.74
NN (73.103.143.129.369)	3315	-	-	0.58	0.12	-
NN (73.105.271.482)	3324	-	-	-	-	'0.52
Triterpenoid, TMS (189.73.375)	3321	-	-	0.20	0.60	-
Lanosterol , TMS	3329	3331	-	-	-	0.53
Triterpenoid, TMS (189.73.143.129)	3338	-	-	0.17	-	-
β-Amyrin, TMS	3341	3345	-	-	-	0.10
α-Amyrin, TMS	3374	3378	-	1.43	-	0.22
C29H48O? (124.229.412.43.55)	3396	-	-	-	-	0.21
Triterpenoid, TMS (189.73.375)	3414	-	-	-	0.11	-
NN (175.117.43.483)	3443	-	-	-	-	0.07
Pinobanksin 3-hydrocinnamate, tri-TMS	3452	3449	-	-	0.55	-
Triterpenol, TMS (199.69.73)	3467	-	-	-	-	0.08
Triterpenoid, TMS (357.73.400.189.131)	3477	-	-	-	-	0.09

Dipterocarpol, TMS	3507	3509	-	-	-	4.32
Triterpenoid, TMS (73.129.189.375)	3507	-	-	-	0.31	-
NN (73.179.573)	3512	-	0.44	-	-	-
NN (73.91.515.79)	3549	-	0.47	-	-	-
Triterpenoid, TMS (73.189)	3555	-	-	-	0.09	-
Triterpenoid, TMS (189.143.73)	3566	-	-	0.18	-	-
Oleanoic acid, di-TMS	3578	3578	-	-	0.08	-
Triterpenoid, TMS (131.215.73)	3593	-	-	-	-	1.42
Triterpenoid, TMS (131.215.73)	3600	-	-	-	-	1.87
NN 73.439.179.294.131.527)	3619	-	0.52	-	-	-
Triterpenoid, TMS (203.189.73.452)	3628	-	-	-	-	1.04
Triterpenoid, TMS (73.189.203.598)	3695	-	-	-	0.14	-
NN (368.73.397.411)	3720	-	-	0.25	-	-
NN (121.73.179.546.573)	3731	-	0.59	-	-	-
NN (531.73.367)	3834	-	0.52	0.52	-	-
1,3-di-*p*-coumaroylglycerol, tri-TMS	3865	3870	-	-	-	1.19
1,3-di-*p*-coumaroyl-2-acetylglycerol, di-TMS	3955	3964	-	-	-	16.73
1-Caffeoyl-3-*p*-coumaroylglycerol, tetra-TMS	4020	4026	-	-	-	1.00
NN (105.73.271.482.194)	4027	-	-	-	-	0.75
2-Acetyl-1-*p*-coumaroyl-3-feruloylglycerol, di-TMS	4136	4139	-	-	-	0.30
2-Acetyl-1-*p*-coumaroyl-3-caffeoylglycerol, tri-TMS	4168	4170	-	-	-	27.16
1-Caffeoyl-3-feruloylglycerol, tetra-TMS?	4172	-	-	-	-	0.94
1-Acetyl-3-caffeoyl-2-feruloylglycerol, tri-TMS?	4200	-	-	-	-	1.22
2-Acetyl-1-caffeoyl-3-feruloylglycerol, tri-TMS	4211	4209	-	-	-	25.87
Sitosterol-3β-*O*-glucoside, tetra-TMS	>4500	4622	-	-	-	0.16
Sesquiterpene & sesquiterpenoids			0.80	23.68	1.00	trace
Triterpenoids			trace	6.66	1.71	11.76
Phenylpropenoids (cinnamic acid derivatives)			30.85	16.82	13.43	3.57
Phenylpropenoid glycerides			-	-	-	74.94
Flavonoids & chalcones			41.28	34.44	79.51	1.38
Aliphatic acids			0.12	1.06	1.29	3.28
Other			17.08	4.48	0.60	3.53
NN			9.87	12.86	2.46	1.53

RI – retention indices, *less than 0.01% TIC, ** component not found, **TMS** – trimethylsilyl derivative of compound, **?** - identified tentatively based on the MS fragmentation patterns, **NN** – component not identified
